# Multilayered genetic safeguards limit growth of microorganisms to defined environments

**DOI:** 10.1093/nar/gku1378

**Published:** 2015-01-07

**Authors:** Ryan R. Gallagher, Jaymin R. Patel, Alexander L. Interiano, Alexis J. Rovner, Farren J. Isaacs

**Affiliations:** 1 Department of Molecular, Cellular & Developmental Biology, Yale University, New Haven, CT 06520, USA; 2 Systems Biology Institute, Yale University, West Haven, CT 06516, USA

## Abstract

Genetically modified organisms (GMOs) are commonly used to produce valuable compounds in closed industrial systems. However, their emerging applications in open clinical or environmental settings require enhanced safety and security measures. Intrinsic biocontainment, the creation of bacterial hosts unable to survive in natural environments, remains a major unsolved biosafety problem. We developed a new biocontainment strategy containing overlapping ‘safeguards’—engineered riboregulators that tightly control expression of essential genes, and an engineered addiction module based on nucleases that cleaves the host genome—to restrict viability of *Escherichia coli* cells to media containing exogenously supplied synthetic small molecules. These multilayered safeguards maintain robust growth in permissive conditions, eliminate persistence and limit escape frequencies to <1.3 × 10^−12^. The staged approach to safeguard implementation revealed mechanisms of escape and enabled strategies to overcome them. Our safeguarding strategy is modular and employs conserved mechanisms that could be extended to clinically or industrially relevant organisms and undomesticated species.

## INTRODUCTION

Since the advent of genetic engineering (1), genetically modified organisms (GMOs) have enabled functional testing of mutations and production of valuable pharmaceutical or industrial compounds (2). Advances in synthetic biology have led to GMOs with increasingly complex functions including production of fuels and medicines (2), and genetic circuits that can sense and respond to changing environments (3). As sophisticated GMOs expand to applications in open systems such as environmental (4) or clinical settings (5), there is a growing need for intrinsic biocontainment strategies—robust genetic safeguards that conditionally restrict the host cell's viability to defined environments (6). Specifically, an intrinsic biocontainment strategy able to restrict growth to environments containing synthetic small molecules could prevent a GMO's dissemination and enhance its safety.

Prior strategies for biocontainment are based on designs to control cell growth by engineered auxotrophy (7), essential gene regulation (8) or toxin expression (9,10). While the best-performing safeguards reach the 10^−8^ NIH standard (11) for escape frequency of recombinant microorganisms (12,13), each approach carries risk. Auxotrophy can be complemented by metabolite cross-feeding (14) or by environmental availability of essential small molecules, yielding strains that grow in rich media and natural environments. Leaked expression of essential genes can permit viability (8) and mutations lead to loss of toxins (15). Attempts to implement redundant safeguards reduce the risk of escape, but at the price of decreased fitness (16,17), leading to a growth advantage for escaping mutants.

**Figure 1.**
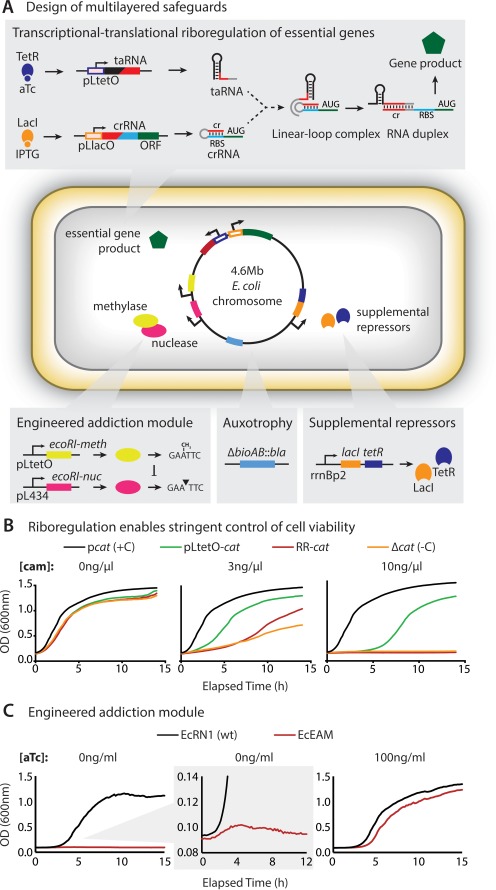
Design of multilayered genetic safeguards: riboregulation, engineered addiction, auxotrophy and supplemental repressors. (**A**) Riboregulation system: pLtetO promoter (19), repressed by TetR and induced by aTc, drives *trans*-activating (taRNA); pLlacO promoter, repressed by LacI and induced by IPTG, drives *cis*-repressed (crRNA) and essential gene. crRNA and taRNA fold through a linear loop intermediate to reveal the crRNA's RBS permitting expression (green). Supplementary TetR (purple) and LacI (green) are constitutively expressed from the genome. Carbenicillin resistance gene (*bla*) replaces *bioAB*, resulting in biotin autotrophy (blue). Constitutive EcoRI nuclease (magenta) enables inducible cell killing in the absence of EcoRI methylase (yellow), which is controlled by aTc. (**B**) Riboregulation system controls cell viability. Chloramphenicol (cam) resistance of strains carrying *cat* gene regulated in different ways, all grown without inducers. Constitutive (black, + control); pLtetO (green, non-riboregulated control); riboregulated (red); no *cat* (orange, - control). (**C**) Kinetic growth curves demonstrate dependence of engineered addiction system (strain EcEAM) on aTc.

We propose that genetic safeguards possess three crucial properties: (i) low escape frequency, (ii) robustness and (iii) modularity. Safeguards with low escape frequency will prevent the rise of mutants escaping defined media and limit growth in the wild. Robust safeguards retain wild-type levels of fitness while also maintaining containment in diverse growth conditions. This crucial requirement demands that low escape frequency safeguards maintain their performance in rich or diverse environments, where provision of auxotrophic metabolites by other community members is possible. Modularity will allow many different strategies to be combined in one strain enabling multilayered safeguards, or for those safeguards to be transferred to different organisms enabling portability. To satisfy these three requirements, we present a strategy based on the staged introduction of independently acting safeguards that use auxotrophy, engineered riboregulation and engineered addiction to permit robust growth only in defined environments containing synthetic small molecules (Figure 1).

## MATERIALS AND METHODS

### Plasmids—cloning and DNA synthesis and assembly

Basic molecular biology techniques were used in plasmid construction. Riboregulated essential gene plasmids (18) were constructed by amplifying genes from *Escherichia coli* using primers to add KpnI and HindIII restriction sites (Supplementary Table S1). Those fragments were cloned between KpnI and HindIII sites in the pZE21Y12a12C vector (Supplementary Figure S1). For all cloning, insert amplicons were purified using spin columns (QiaGen), digested with restriction endonucleases (NEB), agarose gel purified, extracted (QiaGen), ligated (Quick Ligase, NEB), then transformed by electroporation with parameters 1800 V; 25-μF capacitance; 200-Ω resistance; in 1-mm cuvettes (Bio-Rad). The pBAD21G plasmid was created by cloning the p_ara_BAD promoter amplified from pBAD-HisB (Invitrogen) between XhoI and KpnI in plasmid pZE21G (18). Toxin gene plasmids were created by cloning into KpnI- and HindIII-cut pBAD21G or by Gibson Assembly (NEB) into the same vector. For Gibson Assembly (20), the cloning vector was linearized by amplification using primers annealing near KpnI and HindIII sites. Toxin inserts were amplified using primers that added homologies to the vector termini; these homology arms were designed to anneal to the vector with a *T*_m_ = 60°C (∼25 bp). Toxin genes were either amplified from the *E. coli* chromosome or were synthesized in codon-optimized form (gBlocks, IDT) (Supplementary Table S2). Supplemental repressor plasmids were made by Gibson Assembly (20) into the pBAD21G vector using *lacI* or *tetR* genes amplified from *E. coli* and using synonymously recoded fragments obtained from IDT. Polymerase chain reaction (PCR) reactions were carried out using Hot-Start HiFi Mastermix enzyme (Kapa Biosystems) on a C-1000 thermal cycler (Bio-Rad). The following amplification protocol was used: 3 min at 95°C initial denaturation; 30 cycles of 20 s at 98°C, 15 s at 58°C, 30 s/kb at 72°C; then 3 min at 72°C final extension. Colonies were screened by PCR then confirmed by sequencing.

### Genome integration by dsDNA recombination

Double-stranded DNA recombination was performed using λ-red recombineering as previously described (21). For chromosomal integration of ribo-essential switches and supplemental repressors, dsDNA containing the cassette of interest was amplified from purified plasmids using primers that added 50-bp genome homology arms at both ends, targeting specific genomic loci for integration. These fragments were transformed into a recombination-competent strain and recombinants were isolated by TolC negative selection (see below). Recombination was verified by Sanger sequencing the insertion loci. Essential gene knockout was accomplished by replacing essential gene native sites with the *tolC* gene and selecting for sodium dodecyl sulphate (SDS) resistance. dsDNA cassettes for native site knockout were prepared with 50-bp homologies targeted to the ends of the gene to be replaced.

### Strains and reagents

All plasmids were transformed into Mach1 (NEB; F′ proA^+^B^+^ lacI^q^ ΔlacZM15/fhuA2 Δ(lac-proAB) glnV galK16 galE15 R(zgb-210::Tn10)Tet^S^ endA1 thi-1 Δ(hsdS-mcrB)5) or DH5a (Invitrogen; F- φ80lacZΔM15 Δ(lacZYA-argF) U169 recA1 endA1 hsdR17 (rk−, mk+) phoA supE44 λ- thi-1 gyrA96 relA1) cells. All dsDNA recombination steps were carried out in EcNR1 [MG1655 Δ(ybhB-bioAB)::[λcI857 N(cro-ea59)::tetR-bla]]. All strains were grown in low salt LB-Lennox media (10-g tryptone, 5-g yeast extract, 5-g NaCl in 1-l dH2O) or for auxotrophy experiments in EZ Rich Defined Medium (Teknova) with 0.4% glycerol. Plasmids were maintained using kanamycin at 30-μg/ml final concentration. Recombination-competent strains were grown with 50-μg/ml carbenicillin final concentration. Riboregulators were induced with anhydrotetracycline (aTc), isopropyl thiogalactoside (IPTG) or L-arabinose at final concentrations of 20 ng/ml, 0.1 mM or 0.2%, respectively. The *tolC* gene was selected by growth in 0.005% SDS. Oligonucleotides were obtained from IDT (Coralville, IA, USA) or from WM Keck Oligo Synthesis Resource (Yale University, New Haven, CT, USA).

### Chromosomal modification by ssDNA recombination

To introduce the *lacIq1* allele at the *lacI* locus in safeguard strains, ssDNA recombination was used (22,23). Briefly, we designed a 5′-phosphorothioated 90-bp oligonucleotide targeted to the lagging strand of the replication fork at the *lacI* locus. The center of this oligonucleotide specified the modification to be made (multibase deletion).

### Negative selection

Counter selection for markerless replacement of the *tolC* gene was performed as described previously (24). Briefly, strains were transformed with dsDNAs designed to replace *tolC* with a desired cassette. After dsDNA recombination and recovery, cultures were incubated for 10 h with purified colicin E1 protein. Counter-selected cultures were then plated on solid media and single colonies were screened for the expected recombination by PCR or by growth in SDS to confirm loss of resistance.

### Fitness assays

Multiplex growth assays were conducted in a Biotek spectrophotometric 96-well plate reader (Synergy HT) programmed to measure optical density at 600 nm every 10 min over ≥12 h. Each well was seeded with 150 μl of LB-Lennox media containing a 1:100 dilution of late log phase cells. To compute the maximum doubling time (DT), a Matlab script was used to fit the time-course OD data to a smoothed spline, and to find the growth curve inflection point of that spline.

### Escape frequency assay

Safeguarded strains were grown in permissive conditions to late log, then washed three times with distilled water and diluted over a 10-fold series down to 10^7^-fold. Fifty microliter samples of each dilution were plated on both permissive (+ inducer) and non-permissive (− inducer) solid media. Plates were grown for 24 h before colonies were counted. Escape frequencies are reported as triplicate results of (colonies on non-permissive plate × dilution)/(colonies on permissive plate × dilution) plus or minus standard deviation.

### Competitive co-culture

The non-safeguard competitor strain was made by introduction of a marker allele (premature stop codon in *lacZ*) in the EcNR1 ancestor strain. A ribo-essential *ribA* safeguard strain was marked by integration of a kanamycin resistance cassette. The competitor strain and the safeguard strain were grown separately in permissive conditions to late log, then washed three times and resuspended in phosphate buffered saline. Washed cultures were mixed 1:1 by volume, and the mixture was diluted 1:100 into 5 ml of permissive (aTc, IPTG, carbenicillin) or non-permissive (carbenicillin only) media. Every 12 h for 60 h, each co-culture (permissive or non-permissive) was diluted 1:100 into 5 ml of fresh media. For the permissive co-culture (grown +aTc and +IPTG), at each time step, a 10-fold dilution series was plated on differential permissive media (aTc, IPTG, XGAL, carbenicillin) and the blue/total colony quotient was calculated. This quotient was reported as EcR1rib prevalence. For the non-permissive co-culture (grown −aTc and −IPTG), at each time step, a 10-fold dilution series was plated on both non-permissive plates (kanamycin only) and on permissive plates (aTc, IPTG, carbenicillin). Since only EcR1rib escape mutants grow on kanamycin, whereas carbenicillin +aTc +IPTG plates support growth of all cells, we reported [non-permissive Colony Forming Units (CFU)]/[permissive CFU] as the escape mutant frequency.

Using the relative abundances of wild type and contained populations as determined by blue/white colony counts, the mean DT for each population was determined by the following equation:

\begin{equation*} (A_i )\left( {2^{Te/Td} } \right) = (A_f ) \times G .\end{equation*}

In this equation *T_d_* represents mean DT, *T_e_* represents elapsed time (720 min per dilution step), *A_i_* represents relative abundance at the initial time point, *A_f_* the relative abundance at the final time point and *G* represents total growth (using plate-based CFU counts, we find *G* to be ∼100-fold growth at each step, as expected). Solving this equation for *T_d_* yields

\begin{equation*} T_d = \frac{{T_e }}{{\log _2 \left( {\frac{{G \times A_f }}{{A_i }}} \right)}}. \end{equation*}

We compute *T_d_* for the contained (EcR1rib) and competitor subpopulations at each dilution step. Averaging these values allowed us to calculate relative fitness of the contained strain with respect to its ancestor (Supplementary Table S3).

### Whole genome sequencing

#### Sample selection

For each switch class (EcR1rib, EcR1rib+, EcR2nad, EcR1ribR2nad+ and EcTeco), a contained clone and three escaping clones were sequenced. Additionally, the ancestral MG1655, EcNR1 and EcNR1.ΔTolC genomes were sequenced.

#### gDNA prep

Two milliliters of confluent cell culture in LB-Lennox broth were processed with a Qiagen DNeasy Blood and Tissue (cat: 69504) to extract genomic DNA. gDNA quality was assessed on a spectrophotometer (assay for A260/280 ratio between 1.8 and 2.0) and by gel electrophoresis (assay for a tight smear at ∼50 kB).

#### Sequencing

2.5 μg of gDNA, eluted in 50-μl TE pH 8.0, was sent to the Yale Center Genome Analysis for library prep. One to two micrograms of gDNA were sheared to an expected size of 500 bases with Covaris E210 in a covaris microtube (Duty cycle: 5%; Intensity: 3; Cycles per burst: 200; Time: 80 s). Post-shearing cleanup was done with SPRI magnetic beads (Beckman Coulter). QC was then performed on a DNA 1000 bioanalyzer chip. ‘With Bead’ fragment end repair was performed with End Repair enzyme at 20°C for 30 min and purified with a 20% PEG, 2.5-M NaCl solution. ‘With bead’ A-base addition was performed with A-Tailing enzyme at 30°C for 30 min and purified with a 20% PEG 2.5-M NaCl solution. Samples were barcoded with an adapter ligation mix (5-μl 5× buffer; 15-μl Multiplexing Adapter; 5-μl DNA ligase; 5-μl nuclease free water). Ligation was purified with a 20% PEG, 2.5-M NaCl solution. Samples were PCR enriched (26-μl DNA; 30-μl KAPA HiFi Mastermix; 2-ul 25-μM PCR Primer MP1.0; 2-μl 25-μM barcode-specific primer). Samples were loaded onto a lane of an Illumina HiSeq 2000 for 76-base paired-end reads, providing an average genome coverage of 132X.

#### Data analysis

Raw FASTA reads were sorted by barcode into individual forward and reverse sample files. After this processing, reads were exactly 76 bases long. Paired-end reads were aligned to an MG1655 reference sequence (U00096) with Bowtie2. This reference sequence had been indexed with Bowtie2-build. The aligned SAM file was converted to a BAM file with Samtools view. The BAM file was sorted and then indexed with Samtools 0.1.18. Single-nucleotide polymorphisms (SNPs) and small insertions/deletions were called from the sorted BAM file with Freebayes, using default parameters. The resulting calls were initially filtered for those with a root mean square Phred Quality Score of >20. For each sample, SNPs that were also present on the EcNR1 ancestor sequence were filtered out. After these filters, strain-specific mutations were identified. Given the manageable quantity of SNPs, these were then visually vetted using Integrative Genomics Viewer (IGV) to remove false positives. SNPs were only retained if coverage at a site was >10, and if the SNP was represented on >2 plus-strand reads and >2 minus-strand reads.

## RESULTS

### Metabolic auxotrophs fail in rich and diverse media

We first replaced *bioA* and *bioB* genes with the *bla* resistance marker to create a safeguard layer based on biotin auxotrophy, which mimics prior efforts based on metabolic auxotrophy. In LB, this strain grew with a 56 min DT, equal in fitness to its *E. coli* MG1655 ancestor (Supplementary Figure S2 and Supplementary Table S4). In Rich Defined Media, this strain was dependent on biotin supplementation for viability. However, biotin supplementation was not required in blood- and soil-based media (Supplementary Figures S3 and S4), showing that environmental cross-feeding can compromise biosafety strategies based on auxotrophy alone. This result motivates the development of synthetic auxotrophs whose missing essential gene functions cannot be complemented by metabolic cross-feeding, rather only by exogenous supply of synthetic small molecules.

### Riboregulated essential gene strains are dependent on synthetic small molecules

To develop a synthetic auxotroph safeguard resistant to environmental cross-feeding, we began by constructing strains dependent on exogenous supply of synthetic small molecules for essential gene expression. To identify a regulatory strategy capable of robust induction and low basal expression (leakage) necessary to control viability, we compared the performance of an engineered promoter (19) and an engineered riboregulator (18,25). We analyzed growth of strains using constitutive (p*cat*), inducible (pLtetO-*cat*) or riboregulated (ribo-*cat*) control of the antibiotic resistance gene *cat* in the absence of small molecular inducers. We found that coupled transcriptional and translational control of engineered riboregulators confers the stringent expression required to control cell viability (Figure 1B and Supplementary Figure S5).

**Figure 2.**
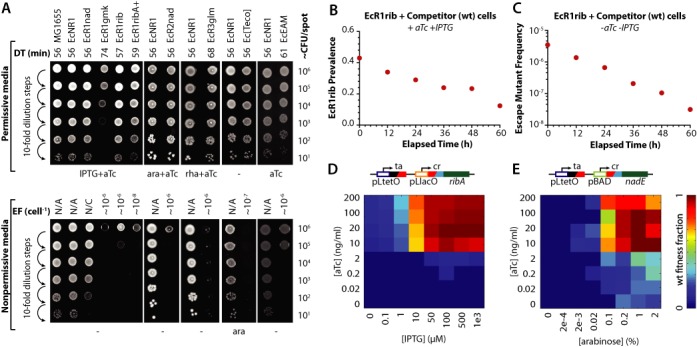
Characterization of ribo-essential and toxin safeguards. (**A**) Dilution series (by row) of strains carrying different riboregulated essential genes or toxins (by column) growing on permissive and non-permissive solid media. Ribo-essential strains based on *ribA, nadE* or *glmS*, respectively, require IPTG, arabinose or rhamnose plus aTc to grow. Doubling time (DT) and escape frequency (EF) are given for each strain. EAM-based safeguards depend on aTc. N/C denotes not contained, implying EF cannot be calculated. (**B**) Riboregulated *ribA* strain grown in mixed culture with ancestral strain in permissive media (+aTc, +IPTG). (**C**) Mixed culture experiment in non-permissive media (-aTc, -IPTG) compares fitness of ancestor and escape mutants. (**D**) Fitness heat map of riboregulated *ribA* (induced by IPTG and aTc) grown with arabinose or (**E**) *nadE* (induced by arabinose and aTc) grown with IPTG in strain EcR1ribR2nad+. Color represents maximum doubling time, as a fraction of wt.

We next adapted riboregulation for native essential genes to create synthetic auxotrophs whose viability could be controlled by synthetic small molecule inducers. From ∼300 essential genes in *E. coli* (26), we selected 13 that span a broad range of cellular processes (Supplementary Table S5). We focused on genes whose function could not be complemented by cross-feeding, either due to limited permeability of the gene's small molecule product (e.g. *ribA*) (27), or because the gene's product carries out an essential intracellular enzymatic function (e.g. *glnS*). We excluded essential genes for which knockout by a selectable cassette would cause polar effects. We cloned essential genes into a riboregulator vector (ribo-essential cassettes) (Supplementary Figure S1), then integrated ribo-essential cassettes in the *E. coli* chromosome using λ Red recombination and markerless positive/negative *tolC* selection. Knockout of essential gene native sites was successful for nine of the 13 cases (*ribA, adk, pyrH, glmS, gmk, nadE, acpP, tmk, lpxC*); these strains failed to form colonies on non-permissive media (lacking aTc and IPTG), but formed colonies on permissive media (containing aTc and IPTG) (Figure 2A). In contrast to the biotin auxotroph, non-permissive blood- and soil-based media did not support growth of ribo-essential strains (Supplementary Figures S3 and S4). Importantly, these results show that ribo-essential regulation permits creation of auxotrophic strains that can only be rescued with synthetic small molecules rather than by supply of missing metabolites.

To determine the fitness cost of ribo-essential expression, we obtained kinetic growth curves for ancestral and ribo-essential strains in the presence or absence of inducers. Several strains possessed near wild-type fitness when grown in permissive media: 56 min per doubling compared to 57 for EcR1rib, 57 for EcR1adk and 56 for EcR1glmS (Figure 2A and Supplementary Table S4). Other ribo-essential strains (e.g. EcR1gmk, EcR1pyr) displayed a fitness defect compared to the ancestor. We also examined the frequency of escape mutants by plating serial dilutions of ribo-essential strains on permissive and non-permissive solid media. These experiments revealed an escape frequency of ∼10^−6^ for all ribo-essential strains (Figure 2A and Supplementary Table S4). Together, these experiments validate essential gene riboregulation as a safeguard with low escape frequencies and growth rates on par with wild-type ancestors.

To examine fitness and escape frequency of ribo-essential strains in a competitive environment, we mixed EcR1rib with its ancestor (EcNR1) and performed a competitive growth experiment in liquid media (Figure 2B and Supplementary Figure S6). Sixty hours of growth in permissive media with six 100-fold dilutions revealed an 8% fitness defect compared to the ancestral strain (Supplementary Table S3). A complementary experiment mixed ancestral and ribo-essential cells in non-permissive media. Accumulation of escape mutants was not observed; instead their frequency in the population fell 100-fold over 60 h, suggesting the strength of selection for escape mutants in non-permissive media is small (Figure 2C) and that escape mutants are outcompeted by wild-type strains.

### Higher-order combinations of safeguards reduce escape frequency

We then attempted to reduce the frequency of escape by creating strains with higher-order combinations of safeguards. First, we created a strain with two ribo-essential cassettes; however, this modification gave no improvement in escape frequency (Supplementary Table S4). We conducted whole genome sequencing (Supplementary Methods) to identify the genetic basis of escape and found recurring frameshift mutations at a known mutable site in the *lacI* gene (28) (Supplementary Table S6). Introduction of these mutations in a contained background using multiplex automated genome engineering (MAGE; (23,29)) leads to escape. However, in mutants isolated by growth on non-permissive media, containment could be restored by transformation with episomal *lacI* (Supplementary Figure S7). We hypothesized that mutations compromising the LacI repressor deregulated both ribo-essential genes, but that intact copies of *lacI* restored containment. By providing supplemental repressors either episomally or chromosomally, the escape frequency of EcR1rib was reduced to ≤9.9 × 10^−8^ (Supplementary Table S4). Finally, increasing LacI expression using the *lacIq1* allele (30) diminished leaked viability in non-permissive media (Supplementary Figure S8). The enhanced riboregulated *ribA* strain with supplemental repressors and *lacIq1* (EcR1rib+) possessed an escape frequency of 4.6 × 10^−8^ (Supplementary Table S4).

**Figure 3.**
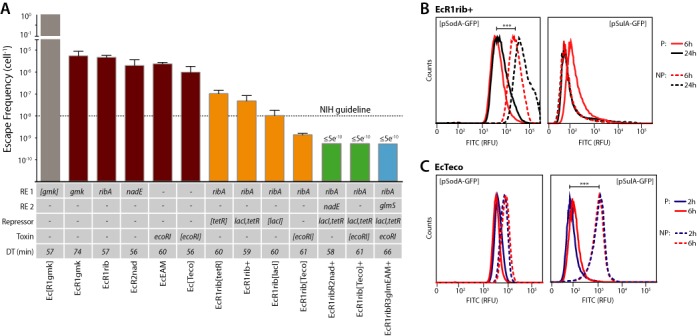
Layering of multiple safeguards reduces escape. (**A**) Escape frequencies (*n* = 3, ±SD) on solid media for strains containing one (red), two (orange), three (green) or four (blue) layers of genetic safeguards. NIH guidelines for work with engineered microorganisms advise a 10^−8^ escape frequency (11) (dotted horizontal line). Strains are characterized by ribo-essential gene (RE 1 or 2), presence of supplemental repressors, presence of a toxin and doubling time (DT) in minutes. Plasmid-based ribo-essential *gmk* safeguard (gray) does not confer containment. Square brackets denote plasmid-based constructs. Limit of detection for solid media is ∼5 × 10^−10^. Flow cytometry shows fluorescence from *sodA* and *sulA* promoters in EcR1rib+ (**B**) or EcTeco (**C**) when grown in non-permissive media (dashed) versus permissive (solid) media for 2 (purple), 6 (red) or 24 (black) h. *** denotes *P* ≤ 0.001, Student's one-tailed *t*-test with Welch's correction.

Repressor supplementation experiments suggested that ribo-essentials could be layered to reduce escape frequency, provided different repressor proteins were used for regulation. Therefore, new riboregulators were built using pAra (arabinose induced) or pRha (rhamnose induced) promoters instead of pLlacO to control *nadE* or *glmS* essential genes, respectively. These new ribo-essential strains (EcR2nad, EcR3glm) were successfully able to link cell viability to the synthetic inducer, demonstrating the flexibility and modularity of the ribo-essential safeguard framework (Figure 2A). We integrated independently riboregulated *ribA* and *nadE* switches to create strain EcR1ribR2nad. This strain required arabinose and IPTG to express the two crRNAs, and aTc to express the common taRNA. Addition of supplemental repressors to create the 3-layer strain EcR1ribR2nad+ (Figure 2D and E) reduced the escape frequency below the detection limit (<5 × 10^−10^) of our solid media assay (Figure 3A and Supplementary Table S4), while maintaining rapid growth (58 min DT compared to 56 for MG1655). During incubation in non-permissive media, CFU counts for this strain on media containing inducers do not drop to zero immediately, rather they decrease gradually over 24 h (Supplementary Figure S8). Importantly, escape frequency assays and long-term challenge on non-permissive media show these cells cannot form colonies on non-permissive media (Supplementary Table S4 and Supplementary Figure S8). This observation suggests that they are not escape mutants and instead represent a non-proliferating persister-like population similar to the low frequency, non-mutant cells that survive antibiotic exposure (31).

### Engineered addiction modules enable construction of bacteriotoxic safeguards

To counter this persister-like population, we constructed and investigated a library of toxin genes for use as bacteriotoxic safeguards (Supplementary Table S7). In proof of concept experiments, cells carrying arabinose-regulated EcoRI endonuclease were killed when induced but grew without a fitness defect (DT equals wild type) when uninduced (Figure 2A). The *E. coli* genome contains 645 EcoRI sites (GAATTC) that are cleavage substrates for the EcoRI endonuclease, overwhelming the cell's ability to repair double-stranded breaks across its chromosome. As a single layer safeguard, this strain (Ec[Teco]) possesses an escape frequency of 9.4 × 10^−7^ (Supplementary Table S4). After transforming the inducible nuclease plasmid to create the 3-layer strain EcR1rib[Teco]+, the frequency of escape fell to 5.6 × 10^−10^ (Supplementary Table S4).

To eliminate the use of antibiotics to retain plasmids and arabinose to express nucleases, which are both important design requirements for intrinsic biocontainment, we built an engineered addiction module (EAM) safeguard (32) that used the cognate methylase of EcoRI endonuclease (33). The EAM reversed regulatory logic so that safeguarded cells would be killed upon removal of an exogenously supplied small molecule (e.g. aTc; Figure 1A). Preliminary experiments showed that aTc-induced EcoRI methylase could protect against the cleavage of GAATTC sites by arabinose-induced EcoRI endonuclease (Supplementary Figure S9). We constructed strain EcEAM by genomically integrating constitutive endonuclease and inducible methylase. This safeguard displayed a delayed induction phenotype, which permitted limited cell growth before rapid killing (Figure 1C, inset). We hypothesize this is caused by the requirement for genome replication to clear GAATTC sites that have been protected by methylation. Importantly, this strain was unviable in the absence of aTc and did not require antibiotic for maintenance of episomal nuclease. As a single-layer safeguard EcEAM displayed 2.4 × 10^−6^ escape frequency and high fitness (61 min DT; Figure 2A).

### Analysis of selected cellular pathways induced by safeguards

To investigate cellular responses in contained strains as they are challenged in non-permissive media we built reporter plasmids containing Green Fluorescent Protein (GFP) fused to promoters of genes (*umuD, polB, dinB, sulA, tisB, sodA, ribA*) previously shown to be implicated in various stress responses (34–36). For EcR1rib+ cells, these experiments revealed 5- and 8-fold increases in the expression of GFP from the *sodA* promoter at 6 and 24 h post inducer deprivation, respectively (Figure 3B), suggesting cells grown in non-permissive media increase expression of superoxide dismutase (*sodA*). Since *sodA* is involved in the response to reactive oxygen species (ROS) (37), this observation suggests contained strains experience ROS stress during inducer deprivation. We observed 8- and 10-fold upregulation of the *sulA* promoter in EcTeco cells (Figure 3C) at 2- and 6-h time points, respectively, which is consistent with reports implicating *sulA* in the response to double-strand DNA breaks (38).

### Long-term, large-scale challenge of multilayered safeguards

**Figure 4.**
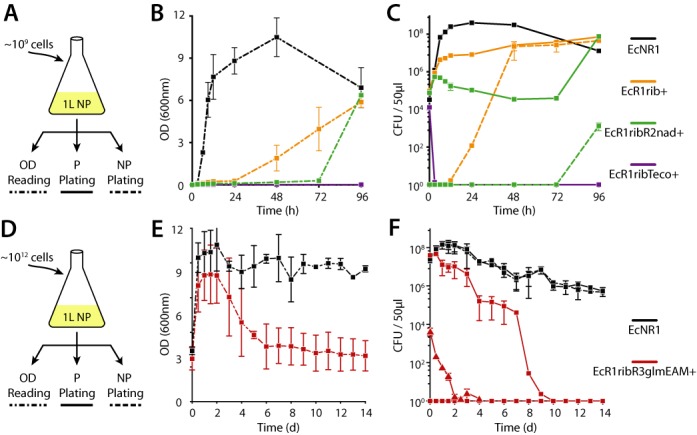
Large volume and long-term challenge of multilayered safeguard strains. (**A**) Strains grown in permissive (P) media to ∼10^9^ cells then challenged over 96 h in 1 l of non-permissive (NP) media, which is monitored for cell density (OD) (**B**) and cell viability (CFU) on permissive (solid line) and non-permissive (dotted line) solid plates (**C**). Biological triplicate results for control strain (EcNR1, black), 2-layer strain (EcR1rib+, orange), 3-layer strain (EcR1ribR2nad+, green) and 3-layer bacteriotoxic strain (EcR1rib[Teco]+, purple) plotted. (**D**) Control (EcNR1, black) and 4-layer safeguard (EcR1ribR3glmEAM+, red) strains grown in permissive, then challenged in 1 l of non-permissive media. (**E**) Strains were monitored by OD readings (**F**) and by plating on permissive or non-permissive solid media over 14 days (squares, ∼10^12^ cell inoculum) or 5 days (triangles, ∼10^8^ cell inoculum). No colonies were observed on NP media for the 4-layer strain.

To analyze the stability of our safeguards over long-term culture, we passaged single- and multilayer strains in permissive media over 110 generations (6 days). Daily plating revealed that escape frequencies for each strain remained stable over time at or near values reported from single time-point escape frequency assays (Supplementary Figure S10 and Supplementary Table S4). To analyze escape and persistence in large cell populations over extended time periods, we inoculated flasks containing 1 L of non-permissive LB with ∼10^9^ CFU of safeguarded strains (Figure 4A–C). Over 4 days of incubation, we monitored cell populations in the flask by measuring OD, and by plating samples on permissive and non-permissive media. Permissive plate counts revealed gradual growth and robust persistence for the 2-layer strain EcR1rib+. Since the inoculum CFU was significantly larger than the 4.6 × 10^−8^ escape frequency of this strain, we detected a proliferating population of escapers within 24 h. CFU counts for 3-layer EcR1ribR2nad+ initially dropped. While the escape frequency of this strain suggests that an escape mutant was not present in the initial inoculum, an escaping population appeared after 72 h. Whole genome sequencing of 3-layer escape mutants revealed deregulating mutations in AraC (39,40) and in the crRNA governing *ribA* (Supplementary Table S6). In escape mutants from Ec[Teco] and EcR1ribR2nad+, sequencing also revealed identical mutations in the mismatch repair (MMR) gene *mutS* (Supplementary Table S6). These experiments highlight a persistent cell population that, while unable to form colonies on non-permissive media, can survive inducer deprivation for ribo-essential strains grown in liquid culture. We hypothesize persistent cells tolerate the stress of inducer deprivation and give the population more opportunities to sequentially defeat safeguards, leading to eventual escape. In contrast to another 3-layer strain (EcR1rib[Teco]+), CFU counts dropped below detectable levels within the first 12 h and remained undetectable for the duration of the experiment. We hypothesize the bacteriotoxic EcoRI safeguard degrades the host genome, preventing persistence so that an escaping population is not observed during the time course of this experiment.

Prior work indicated that *glmS* deficiencies have a bacteriotoxic effect (41), suggesting that a *glmS* synthetic auxotrophy could be used alongside *ribA* and EAM safeguards to counter persistence. We placed *glmS* under the control of a rhamnose- and aTc-induced riboregulator and used this new ribo-essential to create a 4-layered strain (EcR1ribR3glmEAM+). We inoculated 1 L of non-permissive LB with 7.9 × 10^11^ CFU of this strain and incubated for 2 weeks (Figure 4D–F and Supplementary Figure S11). On permissive solid media, viable CFU counts for the 4-layered strains fell ∼10^6^-fold over the first 8 days compared to ∼2-fold for the EcNR1 control. No CFUs were observed on non-permissive media at any time point. Moreover, pelleting then plating the full flask volume on permissive media at day 14 revealed no CFUs, indicating an escape frequency of <1.3 × 10^−12^ (<1/(7.9 × 10^11^)). Since the nuclease-based EAM requires genome replication for methylated sites to be lost, we conducted another experiment with ∼10^8^ CFU inoculum (Figure 4F; red triangles). We hypothesized lower cell density would reveal the dynamics of growth termination, and lead to more rapid outgrowth and faster killing of contained cells. Consistent with our hypothesis, permissive plate CFU counts fell ∼10^4^-fold in 2 days. Taken together, these large-scale experiments show the progressive improvement in containment as additional and distinct safeguard layers are added, culminating in active termination of the inoculum population.

## DISCUSSION

This work describes the implementation and advantages of multilayered genetic safeguards in *E. coli*, whose design is inspired by natural mechanisms of growth regulation. Because at least two independently acting regulatory pathways limit growth and division in animal cells (42), two or more mutations are required for tumorigenesis (43). Similarly, by integrating independently acting safeguards we have constructed strains that must overcome multiple barriers to escape engineered limits on growth and division. We employed safeguards based on auxotrophy, independent essential gene riboregulation, repressor supplementation, and engineered addiction. We characterized these safeguards individually and in combinations to show that they can be integrated in multilayered strains that exhibit limited fitness costs and reduced escape frequency, even when one layer has been compromised. Our multilayered approach employs different modes of action to address the shortcomings inherent to each individual safeguard. The EAM presented here could be applied in other organisms, or could be expanded by use of other previously characterized restriction-modification enzyme pairs (44), provided that they are compatible with native methylation patterns. Importantly, by demonstrating the modularity of ribo-essential regulation through use of several essential genes (*ribA, nadE, glmS, gmk*) and inducible promoter (pLtetO, pLlacO, pAra, pRha) combinations, this work establishes a portable framework for engineering safeguards in other model or undomesticated microorganisms and potentially across members of a microbial community (45). Leveraging the modularity of this approach, future work to engineer riboregulator promoters that use native *cis*-acting elements could prevent the potential loss of natural essential gene regulatory functions.

Probiotic natural isolates (46), and GMOs engineered for delivery of therapeutic DNAs (47), RNAs (48) or proteins (49) to animal cells have already been demonstrated. However, their potential use in open systems such as in human (e.g., probiotic) or environmental applications (e.g., bioremediation) demands safeguard strategies that restrict their growth outside the site of delivery. The frequency of escape mutants must be low enough such that a mutant is unlikely to exist in the population demanded by an application while the strain's fitness must be high enough to ensure execution of its task. For instance, live bacterial vaccines (50,51) or oncolytic therapies delivered by live GMOs (52,53) inoculate with ∼10^6^–10^7^ CFU, well below the escape frequency demonstrated by our best strains. Recent work showing aTc-dependent control of a synthetic gene network in a mammalian gut commensal suggests that regulation of our safeguards is possible *in vivo* (54). Future work to examine extremely large populations of safeguarded cells (>10^12^) could reveal novel escape mechanisms not captured by this study and motivate creation of strains with >4 safeguards. Furthermore, future work could extend these safeguards to previously described strains, permitting safe and secure large-scale bioremediation (4) and therapeutic applications (5,46).

## SUPPLEMENTARY DATA

Supplementary Data are available at NAR Online.

SUPPLEMENTARY DATA
