# Supplementary material for: Multilayered genetic safeguards limit growth of microorganisms to defined environments
Source: Nucleic Acids Res. 2015 Jan 7;43(3):1945–54. doi: 10.1093/nar/gku1378 (PMC4330353; doi:10.1093/nar/gku1378)
Supplement: SUPPLEMENTARY DATA [file supp_43_3_1945__index.html]

Multilayered genetic safeguards limit growth of microorganisms to defined environments — Multilayered genetic safeguards limit growth of microorganisms to defined environments — Multilayered genetic safeguards limit growth of microorganisms to defined environments — SUPPLEMENTARY DATA 

# Multilayered genetic safeguards limit growth of microorganisms to defined environments

## SUPPLEMENTARY DATA

**Files in this Data Supplement:**

- SUPPLEMENTARY DATA
