# Supplementary material for: Multilayered genetic safeguards limit growth of microorganisms to defined environments
Source: Nucleic Acids Res. 2015 Jan 7;43(3):1945–54. doi: 10.1093/nar/gku1378 (PMC4330353; doi:10.1093/nar/gku1378)
Supplement: SUPPLEMENTARY DATA [file supp_gku1378_nar-03386-h-2014-File006.pdf]

Supplementary Materials for:

Multilayered genetic safeguards limit growth of microorganisms to synthetic environments

Ryan R. Gallagher,[1,2*] Jaymin R. Patel,[1,2*] Alexander L. Interiano,[1] Alexis J. Rovner,[1,2] Farren J. Isaacs[1,2†]

[1]Department of Molecular, Cellular & Developmental Biology, Yale University, New Haven, CT, 06520 USA
[2]Systems Biology Institute, Yale University, West Haven, CT, 06516 USA
*These authors contributed equally to this work.
[†]Correspondence to: farren.isaacs@yale.edu

Supplementary Figures 1-11

Supplementary Tables 1-7

Supplementary References

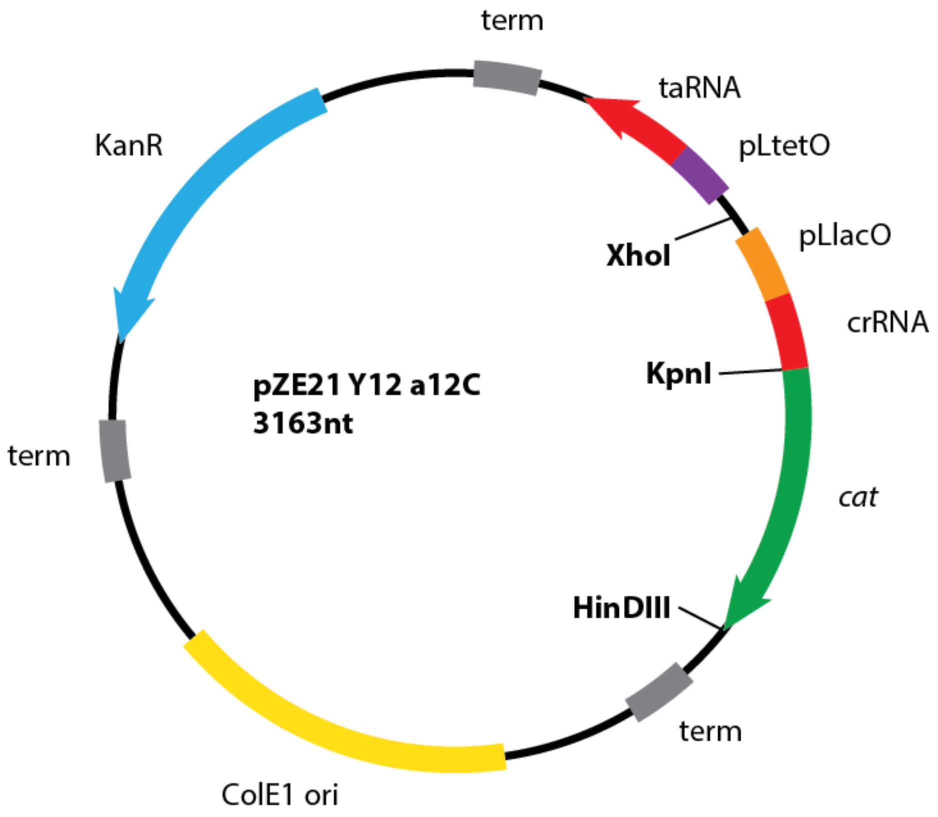

Supplementary Figure 1. Map of the pZE21 Y12 a12C plasmid used to clone essential genes for riboregulated expression. Essential genes were amplified with KpnI and HindIII overhangs for cloning into those unique restriction sites. Primers designed to amplify ribo-essential cassettes from this vector were modified with genome targeting homologies to generate dsDNAs capable of site-specific integration on the *E. coli* chromosome.

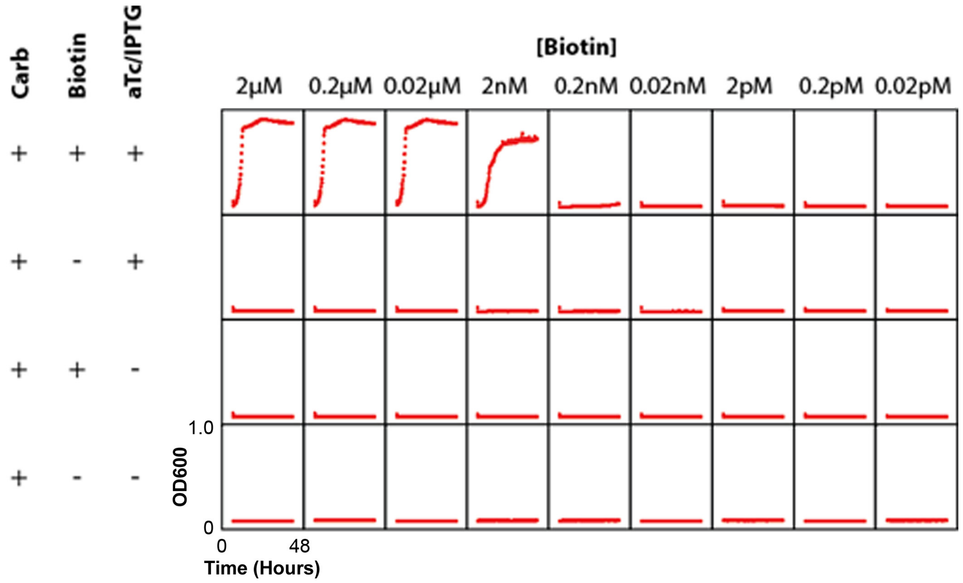

Supplementary Figure 2. Determining biotin supplementation requirements in defined media. EcR1pyr (Supplementary Table 4) was grown to mid-log phase and diluted 1:1,000 into EZ rich defined media (Teknova) containing inducers, biotin at various concentrations, both, or neither. OD600nm was measured on a robotic plate reader over 48 hours to assay growth. Optimal growth was seen at 20nM and strong growth was seen at 2nM biotin supplementation. No growth was observed below 2 nM biotin.

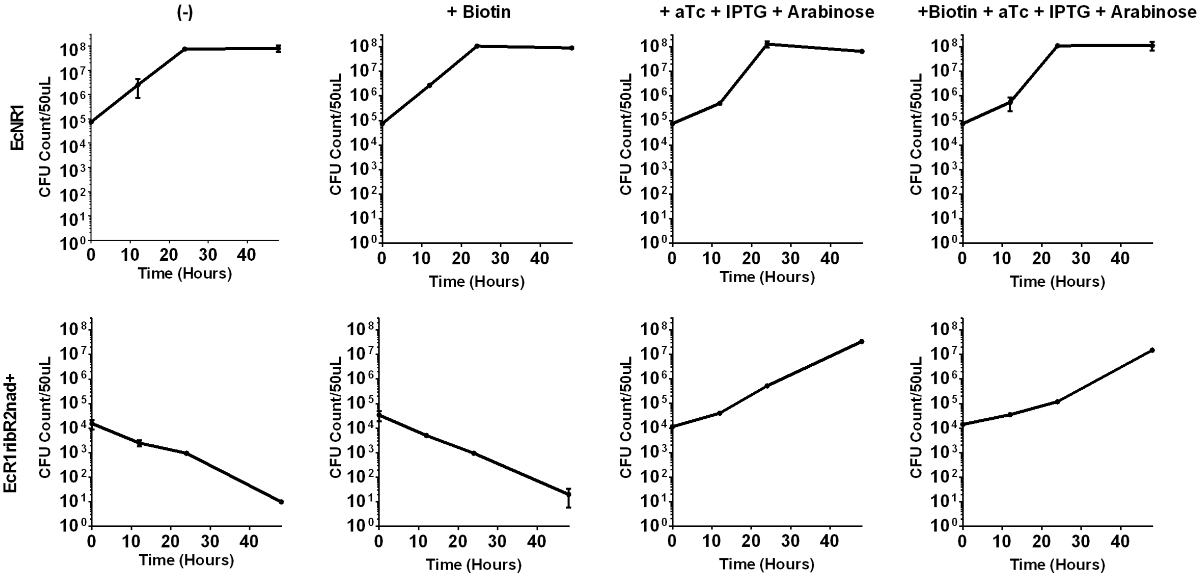

Supplementary Figure 3. Biotin Auxotrophy fails in Sheep Blood. ~10^6 CFUs of the EcNR1 ancestor or the EcR1ribR2nad+ riboregulated strain were inoculated into 3mL defibrinated Sheep Blood (BD cat. 212389). The blood was supplemented with biotin, inducers (aTc, IPTG, arabinose), both, or neither. A 50µL sample was removed every 12 hours for 48 hours for CFU counts. The EcNR1 ancestor, though biotin auxotrophic, was able to proliferate in the blood without biotin supplementation. The riboregulated strain also did not require biotin supplementation, but was reliant on inducer supplementation for viability.

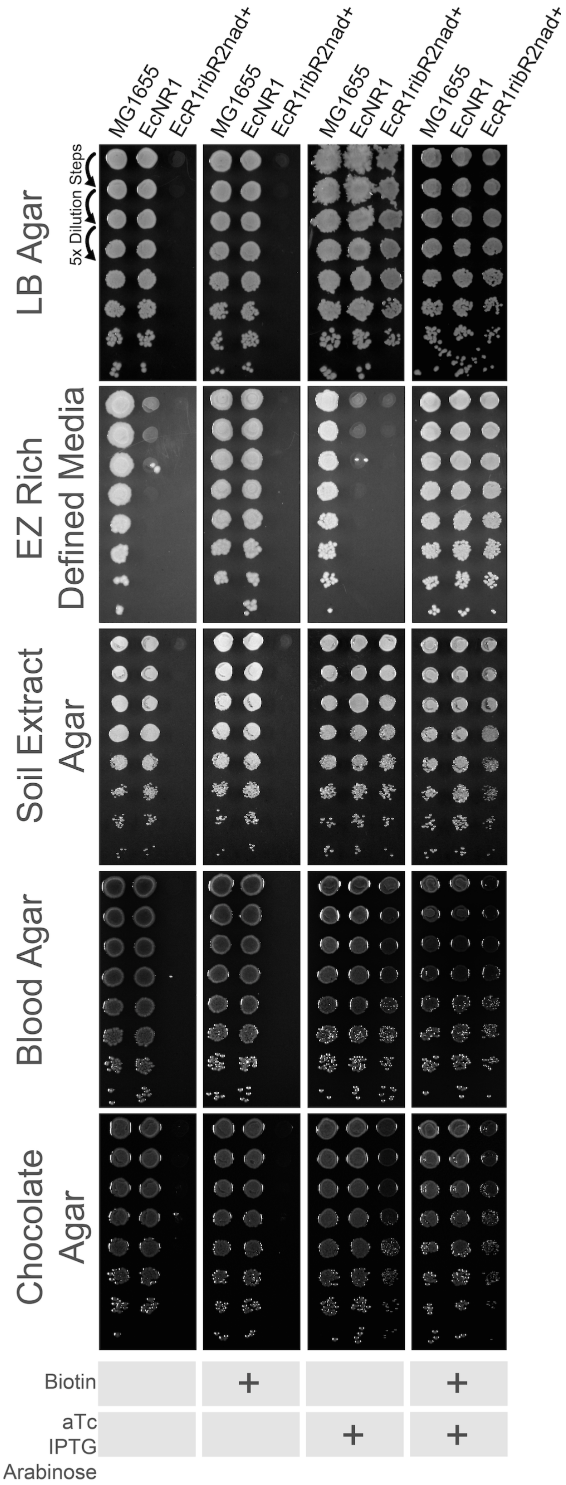

Supplementary Figure 4. Riboregulation-based safeguards are effective across a diverse array of rich environments. Cultures of wild-type MG1655, the EcNR1 biotin-auxotrophic ancestor, and the EcR1ribR2nad+ riboregulated strain were grown to OD 0.8. A 5-fold dilution series was made for each of the strains. With a frogger tool, this series was stamped onto various solid media – LB agar, EZ Rich Defined Media agar (Teknova), Soil Extract agar (Himedia cat. M455), Blood agar (Teknova), and Chocolate agar (Teknova) – and incubated overnight at 34°C. The media was supplemented with biotin, inducers (aTc, IPTG, arabinose), both, or neither. Biotin-auxotrophic strains required biotin supplementation only in the defined rich media. Riboregulated strains required inducer supplementation in all media.

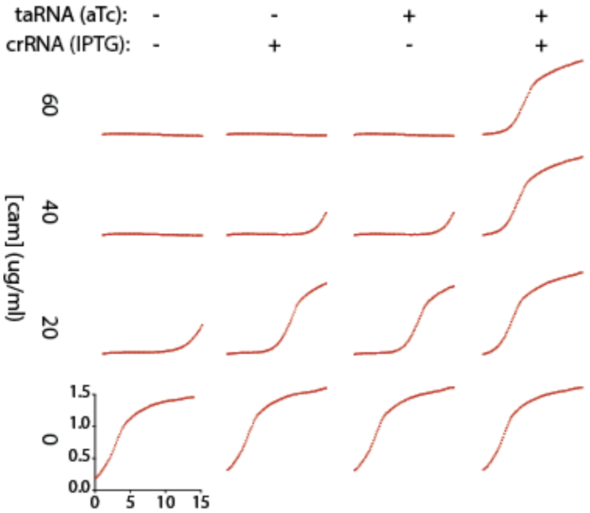

Supplementary Figure 5. Riboregulated *cat* displays AND logic. Kinetic growth curves (x-axis of each subplot: 0-15 hours) monitored by optical density at 600nm (y axis of each subplot: 0-1.5 OD) of strain carrying episomal riboregulated *cat* gene in different concentrations of inducer (aTc for taRNA at 20 ng/ml, and IPTG for crRNA at 100µM – by column) and chloramphenicol (cam – by row). Above a threshold of chloramphenicol concentration, the strain only grows in the presence of both inducers thereby displaying AND logic.

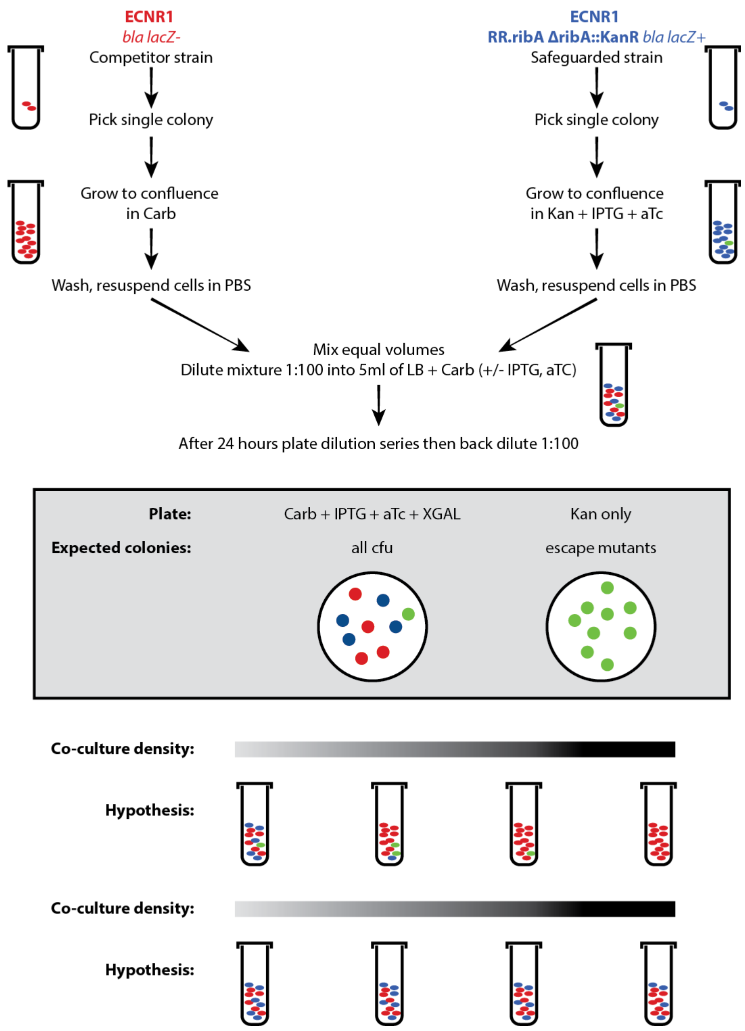

Supplementary Figure 6. Workflow for the competitive co-culture experiment used to determine fitness of safeguarded strain relative to ancestral control. Competitor strain is marked with *lacZ*⁻ allele (1) giving white colonies on IPTG+ XGAL+ media while safeguard strain has *lacZ*⁺ allele giving blue colonies on the same media. This allows the two strains to be discerned on differential permissive media (+IPTG, +aTc, +XGAL). Since only the safeguarded strain carries a kanamycin (kan) resistance gene, only escape mutants can grow on media containing kan without inducers. Both strains carry the carbenicillin (carb) resistance gene *bla* and can therefore both be grown on media containing carb, IPTG and aTc.

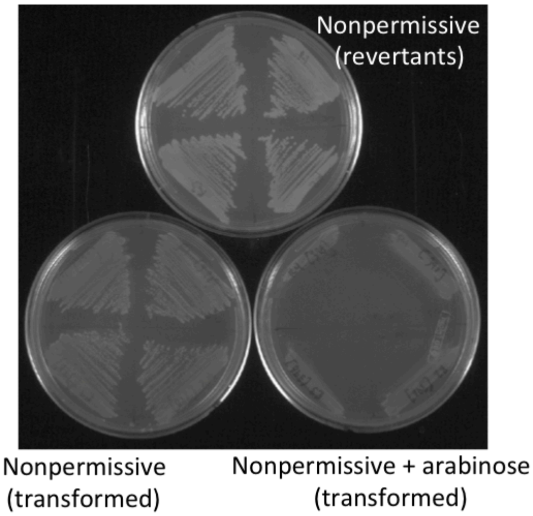

Supplementary Figure 7. *lacI* supplementation can restore inducer dependence in escape mutants. Four independent escape mutants of the *ribA* ribo-essential strain isolated from colonies that arose on nonpermissive media show inducer independence since they are able to grow robustly on media lacking aTc and IPTG (top). Transforming each of these four isolates with a plasmid that carries arabinose-regulated *lacI* results in strains that regain inducer dependence in the presence of arabinose (bottom). This result suggests that mutations in *lacI* are an important mode of escape.

(A)

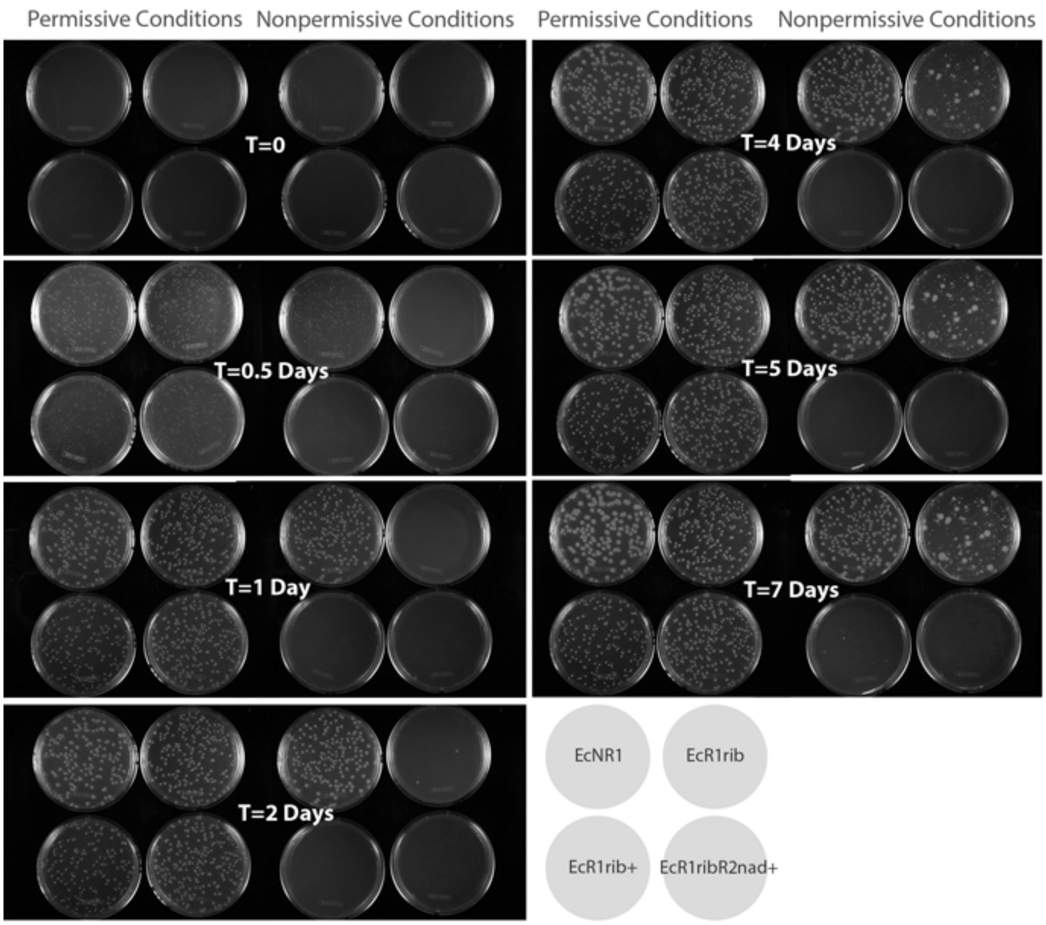

(B)

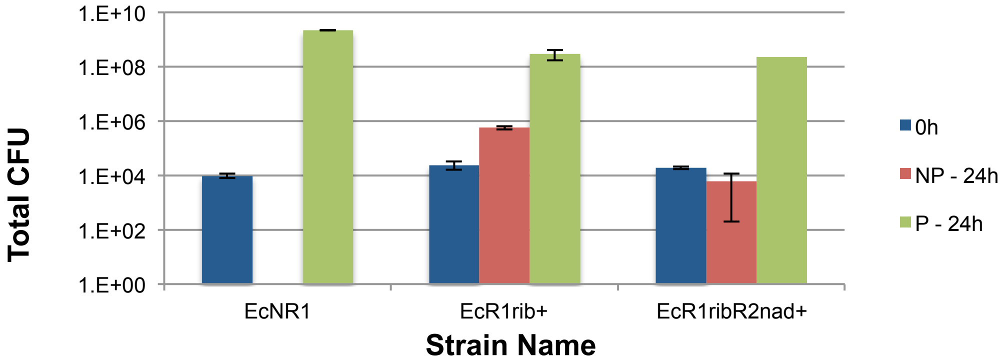

Supplementary Figure 8. Multilayered safeguards reduce leaked viability. (A) Cultures of EcNR1, EcR1rib, EcR1rib+, and EcR1ribR2nad+ were grown to OD 0.8 then diluted 10^5-fold in PBS and spread over permissive media (aTc, IPTG, and arabinose) and over non-permissive media (no inducers). Plates were imaged at various timepoints. In the absence of induction, the EcR1rib strain shows severely attenuated growth, forming extremely slow growing colonies that start to become visible after 48 hours. With supplemental repressors and *lacIq1* (EcR1rib+), uninduced growth is substantially attenuated, and rare microcolonies only become visible at 168 hours. With 2 riboregulated essential genes, no microcolonies are observed. **(B)** EcNR1, EcR1rib+ and EcR1ribR2nad+ were grown in permissive liquid media then plated to count initial CFU (blue), then washed, and inoculated into either permissive (P) or nonpermissive (NP) liquid media. After 24h growth in P (green) or NP (red) liquid media, cultures were plated on permissive solid media to count persistent CFU. EcNR1 in NP media not applicable (N/A).

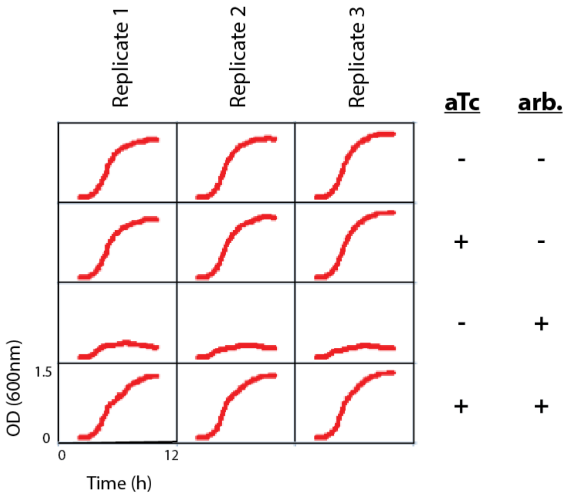

Supplementary Figure 9. EcoRI methylase protects against toxicity of arabinose induced EcoRI nuclease. A strain carrying the arabinose (arb.) induced EcoRI endonuclease plasmid from strain Ec[Teco] is protected by addition of aTc, which induces expression of the EcoRI methylase. Kinetic growth curves depict a 12 hour optical density time course measured over 10 minute intervals at 600nm. All wells seeded 1:100 from an overnight culture into LB with Kanamycin. Arabinose supplied at final concentration of 0.1% w/v, aTc supplied at final concentration of 20ng/ml.

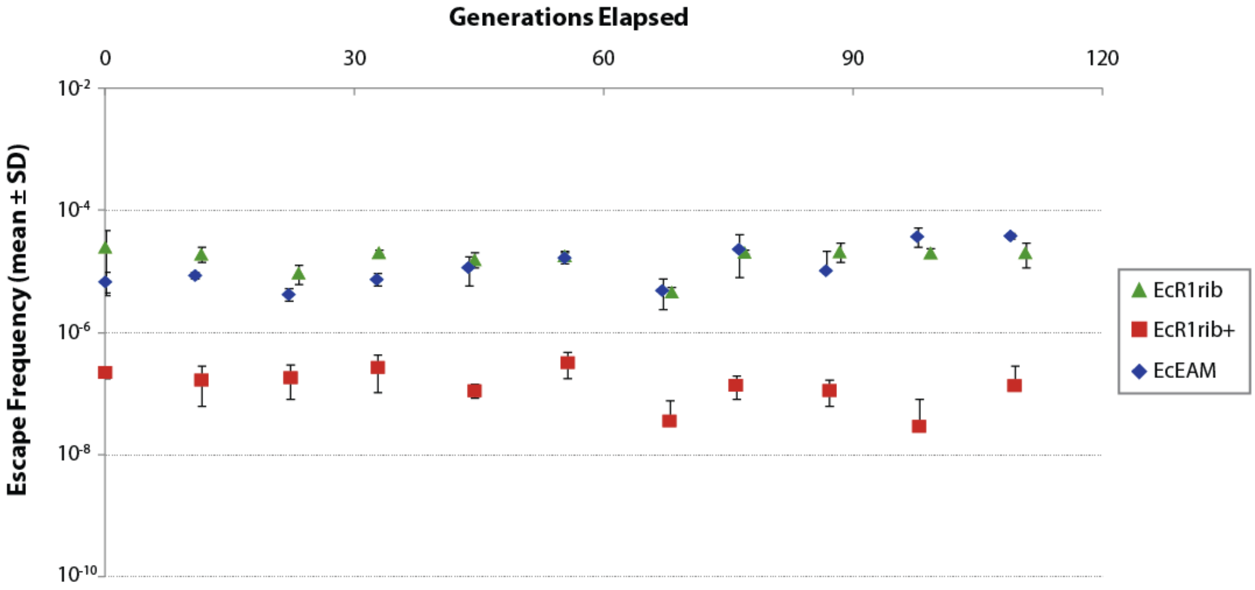

Supplementary Figure 10. Long term continuous culture experiment demonstrates stability of several single- and multi-layer safeguard strains. Triplicate cultures of each strain (single layer EcR1rib and EcEAM, two layer EcR1rib+; refer to Supplementary Table 4) were inoculated into permissive LB media (IPTG, aTc, and arabinose) with carbenicilin to maintain sterility. After overnight growth, each replicate was diluted 1:1,000 into fresh permissive media, and samples were plated on permissive and on nonpermissive media (carbenicilin only). Every twelve hours for 5.5 days (11 time points), cultures were diluted 1:1,000 into fresh permissive media and plated again. Total CFU counts on permissive media were used to calculate elapsed doublings (generations). The quotient of nonpermissive CFU counts divided by total CFU counts were used to calculate the escape frequency of each replicate at each timepoint. Error bars represent the standard deviation of escape frequency measurement for each strain at each timepoint (n=3).

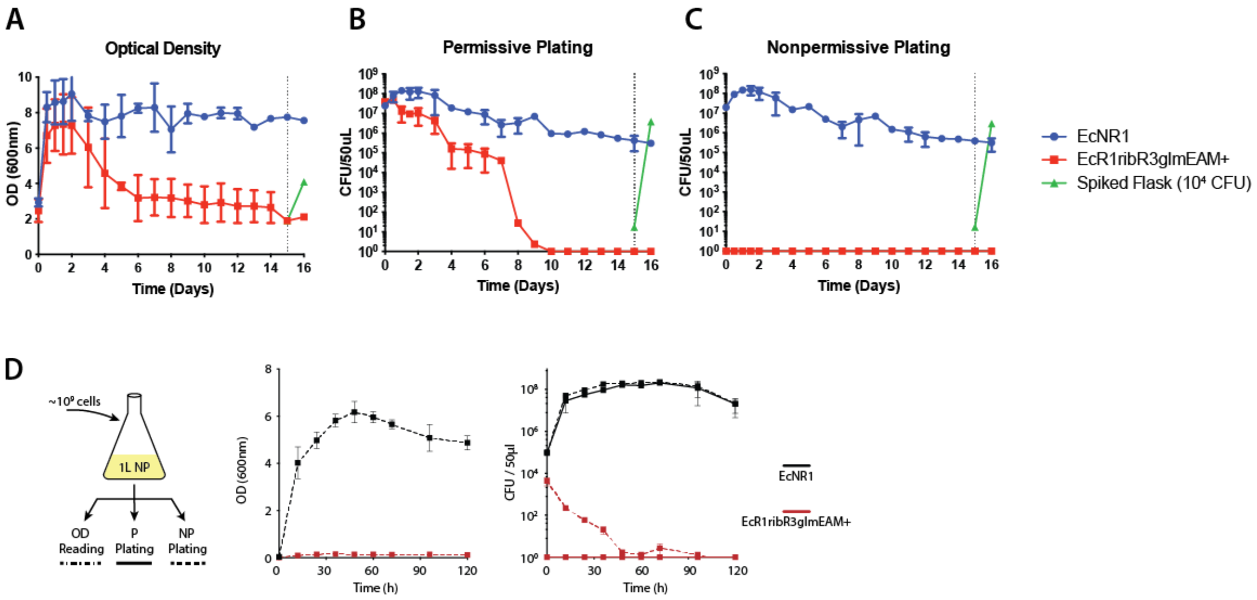

Supplementary Figure 11. Large volume nonpermissive media experiment demonstrates ≤10^{12} escape frequency and active termination of inoculum population. Triplicate flasks containing 1L nonpermissive media (carbenicillin only) were inoculated with 10^{12} CFU of EcNR1 (blue) or EcR1ribR3glmEAM+ (red) and incubated with shaking for 2 weeks. Periodic measurements of optical density **(A)**, CFU on solid permissive media containing (containing carbenicilin, IPTG, aTc, rhamnose, glucosamine; **B**) and CFU on nonpermissive media (carbenicillin only; **C**). No colonies were observed on nonpermissive media. At the end of the experiment, 10^4 CFU of EcNR1 were added to flask containing the 4-layer safeguard strain (green line) to demonstrate the nonpermissive media could support growth of viable cells. Error bars indicate standard deviation of triplicate measurements. **(D)** A smaller-scale experiment using 10^8 CFU of 4-layer safeguard strain (EcR1ribR3glmEAM+) in 1L nonpermissive media (carbenicillin only) shows that with a more dilute inoculum, the engineered addiction system is able to more rapidly terminate safeguarded cells.

Supplementary Table 1. Oligonucleotides used in this study.

Name	Sequence
Cloning the *cat* gene into pLtetO and riboregulation vector	
KpnI.CAT-f	ATTAGGTACCATGGAGAAAAAAATCACTGGATATACC
HindIII.CAT-r	TATAAAGCTTTTACGCCCCGCCCTGCCACT
Cloning essential genes into riboregulation vector	
KpnI.gmk-f	ATT AGG TAC CAT GGC TCA AGG CAC GC
HindIII.gmk-r	TAT AAA GCT TTC AGT CTG CCA ACA ATT TGC
KpnI.tmk-f	ATT AGG TAC CAT GCG CAG TAA GTA TAT CGT CA
HindIII.tmk-r	TAT AAA GCT TTC ATG CGT CCA ACT CCT T
kpn1.acpP-f	TAT AGG TAC CAT GAG CAC TAT CGA AGA ACG C
HindIII.acpP-r	TAT AAA GCT TTT ACG CCT GGT GGC CG
acpP.seq-f	GGT AAG ACC TGC CGG GAT TTA G
acpP.seq-r	CAC AAC TAC ACG ACG CTT AGA C
KpnI.hemA-f	TAT AGG TAC CAT GAC CCT TTT AGC ACT CGG T
HindIII.hemA-r	TAT AAA GCT TCT ACT CCA GCC CGA GGC
hemA.seq-f	GCC AGA ATC TAA CGG CTT TCG
hemA.seq-r	CGT TCA TGC AGG GCT TCC
KpnI.dxr-f	TAT AGG TAC CAT GAA GCA ACT CAC CAT TCT GG
HindIII.dxr-r	TAT AAA GCT TTC AGC TTG CGA GAC GCA
dxr.seq-f	CTG ATG CAG TTC TGA TTT CTT GAA C
dxr.seq-r	GCA TAT CTG ACC TTA TAA AGC CAA CTA C
KpnI.folA-f	TAT AGG TAC CAT GAT CAG TCT GAT TGC GGC
HindIII.folA-r	TAT AAA GCT TTT ACC GCC GCT CCA GAA
folA.seq-f	CAG GGA GAG AGC GTG GAC
folA.seq-r	CGT CGA ACC GGC ATA AGG
KpnI.nadE-f	TAT AGG TAC CAT GAC ATT GCA ACA ACA AAT AAT AAA GGC
HindIII.nadE-r	TAT AAA GCT TTT ACT TTT TCC AGA AAT CAT CGA AAA CG
nadE.seq-f	CCT GTA TGA CGT TTT AAC CAC CA
nadE.seq-r	TGT CAG GCC TAT TCG ACT CC
KpnI.pyrH-f	TAT AGG TAC CAT GGC TAC CAA TGC AAA ACC C
HindIII.pyrH-r	TAT AAA GCT TTT ATT CCG TGA TTA AAG TCC CTT CTT T
pyrH.seq-f	CCC ATC TTG TAA ATT CAG CTA ACC C
pyrH.seq-r	CGT GAC CAA ACT GCC TGC
KpnI.lpxC-f	TAT AGG TAC CAT GAT CAA ACA AAG GAC ACT TAA ACG
HindIII.lpxC-r	TAT AAA GCT TTT ATG CCA GTA CAG CTG AAG G
lpxC.seq-f	GAA TTG ACT GGA ATT TGG GTT TCG
lpxC.seq-r	GTT CAC CTG GCC GGA GAG
KpnI.adk-f	TAT AGG TAC CAT GCG TAT CAT TCT GCT TGG C
HindIII.adk-r	TAT AAA GCT TTT AGC CGA GGA TTT TTT CCA GAT C
adk.seq-f	GCC TTT CTT GAG GCA ATC GC
adk.seq-r	GCC TGA GAT TGC TGA TAA GTT TGC
KpnI.ribA-f	TAT AGG TAC CAT GCA GCT TAA ACG TGT GGC
HindIII.ribA-r	TAT AAA GCT TTT ATT TGT TCA GCA AAT GGC CC
ribA.seq-f	GCC ATT CCG TGA ACG ATC G
ribA.seq-r	CGG CAT TTT GCA TTA TGT CAT TCG
KpnI.glnS-f	TAT AGG TAC CAT GAG TGA GGC AGA AGC CC

HindIII.glnS-r	TAT AAA GCT TTT ACT CGC CTA CTT TCG CCC
glnS.seq-f	CAT CCC CAT AAT CCT TGT TAG ATT ATC A
glnS.seq-r	CTG ATA AGC GTA GCG CAT CAG
KpnI.glmS-f	TAT AGG TAC CAT GTG TGG AAT TGT TGG CGC
HindIII.glmS-r	TAT AAA GCT TTT ACT CAA CCG TAA CCG ATT TTG C
glmS.seq-f	CCC CAC TCT CTA CAA GGC TC
glmS.seq-r	CCG AAG ATG ACG GTT TGT CAC

<u>Integrating ribo-essentials cassettes into genome</u>

1415470(13B).pZ-f	CCTCAACTCAGATTAAAATTCGTTTTGTTCAGTGAATGATCTTGCCGGATCAGGGCTTCCCAACCTTAC
1415471(13B).pZ-r	GAAATCTGAAAGAAATAGCCTGCGTATGGCGCAGGCTATGAACAGTGTGTCGCCTTTGAGTGAGCTGATA
781100(9/10B).pZ-f	AGGCGCTTTTTTGCTATTCAGGCATCCTCAATTTCACTTTGTAAACCTGACAGGGCTTCCCAACCTTAC
781101(9/10B).pZ-r	CTAATTCCACCGACATAGAGTTGCTTGCACAGTAAGCTCTGACGATGTCACGCCTTTGAGTGAGCTGATA
2428900(21B).pZ-f	TTTGCGTAGGGATTTCCTTCCCGCGCATCAATAAAAATGGCGCTGAAAAACAGGGCTTCCCAACCTTAC
2428901(21B).pZ-r	ACGCATTGCCCGATGCCGCAAAGGCATAAAAAGTCGATGGCGTTGAATATCGCCTTTGAGTGAGCTGATA
781100(9/10B).cr-f	AGGCGCTTTTTTGCTATTCAGGCATCCTCAATTTCACTTTGTAAACCTGAGCTAGCATCTCGAGATGCTAGC
1415470(13B).cr-f	CCTCAACTCAGATTAAAATTCGTTTTGTTCAGTGAATGATCTTGCCGGATGCTAGCATCTCGAGATGCTAGC
2428900(21B).cr-f	TTTGCGTAGGGATTTCCTTCCCGCGCATCAATAAAAATGGCGCTGAAAAAGCTAGCATCTCGAGATGCTAGC

<u>Integrating *tolC* at recombinogenic sites</u>

781100(9/10B).TolC-f	AGGCGCTTTTTTGCTATTCAGGCATCCTCAATTTCACTTTGTAAACCTGATTGAGGCACATTAACGCC
781100(9/10B).TolC-r	CTAATTCCACCGACATAGAGTTGCTTGCACAGTAAGCTCTGACGATGTCATCTAGGGCGGCGGATT
2428900(21B).tolC-f	TTTGCGTAGGGATTTCCTTCCCGCGCATCAATAAAAATGGCGCTGAAAAATTGAGGCACATTAACGCC
2428900(21B).tolC-r	ACGCATTGCCCGATGCCGCAAAGGCATAAAAAGTCGATGGCGTTGAATATTCTAGGGCGGCGGATT
1415470(13B).TolC-f	CCTCAACTCAGATTAAAATTCGTTTTGTTCAGTGAATGATCTTGCCGGATTTGAGGCACATTAACGCC
1415471(13B).TolC-r	GAAATCTGAAAGAAATAGCCTGCGTATGGCGCAGGCTATGAACAGTGTGTTCTAGGGCGGCGGATT

<u>Deleting native sites of essential genes with KanR (kanamycin resistance cassette)</u>

acpP.kanR.KO-f	ACA CTA CGA AAA CCA TCG CGA AAG CGA GTT TTG ATA GGA AAT TTA AGA GTC CTG TGA CGG AAG ATC ACT TC
acpP.kanR.KO-r	GAC AAA AAG ATA AAA CTC AGG CGG TCG AAC GAC CGC CTG GAG ATG TTC ACA ACC AGC AAT AGA CAT AAG CGG
hemA.kanR.KO-f	ATG ATG CAA GCA GAC TAA CCC TAT CAA CGT TGG TAT TAT TTC CCG CAG ACC CTG TGA CGG AAG ATC ACT TC
hemA.kanR.KO-r	AGG CTT CAT AGG CGT AAA TGC ACC CTG TAA AAA AAG AAA ATG ATG TAC TGA ACC AGC AAT AGA CAT AAG CGG
dxr.kanR.KO-f	ATC GGC TGG CGG CGT TTT GCT TTT TAT TCT GTC TCA ACT CTG GAT GTT TCC CTG TGA CGG AAG ATC ACT TC
dxr.kanR.KO-r	CTG AAG CCC TAC GCT AAC AAA TAG CGC GAC TCT CTG TAG CCG GAT TAT CCA ACC AGC AAT AGA CAT AAG CGG
folA.kanR.KO-f	GTT TAC GCT TTA CGT ATA GTG GCG ACA ATT TTT TTT ATC GGG AAA TCT CAC CTG TGA CGG AAG ATC ACT TC
folA.kanR.KO-r	AAG ACG CGA CCG GCG TCG CAT CCG GCG CTA GCC GTA AAT TCT ATA CAA AAA ACC AGC AAT AGA CAT AAG CGG
nadE.kanR.KO-f	CAA CGG GTT AGC TTT AAG GAA GTT TTG TCT TTT CTG TCT GGA GGG GTT CAC CTG TGA CGG AAG ATC ACT TC
nadE.kanR.KO-r	CCG GCG TGA ACA AAT TAC TCT TTT TCG CAC AAT CCA ATA TGT GCA AAT TAA ACC AGC AAT AGA CAT AAG CGG
pyrH.kanR.KO-f	TGT CGC TAG TAT TAA TTC ATT TCA ATC GTT GAC AGT CTC AGG AAA GAA ACC CTG TGA CGG AAG ATC ACT TC
pyrH.kanR.KO-r	CCC CCG CGA TAC TTA CGC GGA ATC TTA CCC TTA TTT ATC CAT CAC GGG AAA ACC AGC AAT AGA CAT AAG CGG
adk.kanR.KO-f	CGT TTA TCG CTT TTT CAA AAA ATT CGA CAC ATT TTA AGG GGA TTT TCG CAC CTG TGA CGG AAG ATC ACT TC
adk.kanR.KO-r	CTA ACC CTC TCC CCG AGG GGG CGA GGG GAC TGT CCG TGC GCG CTT TCG AAA ACC AGC AAT AGA CAT AAG CGG
lpxC.kanR.KO-f	CGA ATG TAT AGT ACA CTT CGG TTG GAT AGG TAA TTT GGC GAG ATA ATA CGC CTG TGA CGG AAG ATC ACT TC
lpxC.kanR.KO-r	AGA GAG TGC CAG ATT TGC CAG TCG AAT TTT ATA CGA CAG TAT AAA TGT CGA ACC AGC AAT AGA CAT AAG CGG
ribA.kanR.KO-f	CGT TAT GGC AAA ATA AGC CAA TAC AGA ACC AGC ATT ATC TGG AGA ATT TCC CTG TGA CGG AAG ATC ACT TC

ribA.kanR.KO-r	GCC GGT TAT TTT GCT TCC GGC AAG CAA ATG AAT TAC ACA ATG CAA GAG GGA ACC AGC AAT AGA CAT AAG CGG
glnS.kanR.KO-f	TTA TAA GAT CAT ACG CCG TTA TAC GTT GTT TAC GCT TTG AGG AAT CCA CGC CTG TGA CGG AAG ATC ACT TC
glnS.kanR.KO-r	TTT TAA GTT TCG CTA TGC CGG ATG GGG CGT TTA CGT CGC ATC CGG CAA GGA ACC AGC AAT AGA CAT AAG CGG
glmS.kanR.KO-f	AAA CGG GCA TAC AGG TTG ACC GAC AAC GAT ATA AAT CGG AAT CAA AAA CTC CTG TGA CGG AAG ATC ACT TC
glmS.kanR.KO-r	AAA AAC ATA ACA GGA AGA AAA ATG CCC CGC TTA CGC AGG GCA TCC ATT TAA ACC AGC AAT AGA CAT AAG CGG

Deleting native sites of essential genes with *tolC*

acpP.TC.KO-f	ACACTACGAAAACCATCGCGAAAGCGAGTTTTGATAGGAAATTTAAGAGTTTGAGGCACATTAACGCC
hemA.TC.KO-f	ATGATGCAAGCAGACTAACCCTATCAACGTTGGTATTATTTCCCGCAGACTTGAGGCACATTAACGCC
dxr.TC.KO-f	ATCGGCTGGCGGCGTTTTGCTTTTTATTCTGTCTCAACTCTGGATGTTTCTTGAGGCACATTAACGCC
folA.TC.KO-f	GTTTACGCTTTACGTATAGTGGCGACAATTTTTTTTATCGGGAAATCTCATTGAGGCACATTAACGCC
nadE.TC.KO-f	CAACGGGTTAGCTTTAAGGAAGTTTTGTCTTTTCTGTCTGGAGGGGTTCATTGAGGCACATTAACGCC
pyrH.TC.KO-f	TGTCGCTAGTATTAATTCATTTCAATCGTTGACAGTCTCAGGAAAGAAACTTGAGGCACATTAACGCC
adk.TC.KO-f	CGTTTATCGCTTTTTCAAAAAATTCGACACATTTTAAGGGGATTTTCGCATTGAGGCACATTAACGCC
lpxC.TC.KO-f	CGAATGTATAGTACACTTCGGTTGGATAGGTAATTTGGCGAGATAATACGTTGAGGCACATTAACGCC
ribA.TC.KO-f	CGTTATGGCAAAATAAGCCAATACAGAACCAGCATTATCTGGAGAATTTCTTGAGGCACATTAACGCC
glnS.TC.KO-f	TTATAAGATCATACGCCGTTATACGTTGTTTACGCTTTGAGGAATCCACGTTGAGGCACATTAACGCC
glmS.TC.KO-f	AAACGGGCATACAGGTTGACCGACAACGATATAAATCGGAATCAAAAACTTTGAGGCACATTAACGCC
gmk.TC.KO-f	CGTGATGAAAGCAAAGCCGAGTGGCAAAAACGGAGTCTGCGAGGACGCTTTTGAGGCACATTAACGCC
acpP.TC.KO-r	GACAAAAAGATAAAACTCAGGCGGTCGAACGACCGCCTGGAGATGTTCACTCTAGGGCGGCGGATT
hemA.TC.KO-r	AGGCTTCATAGGCGTAAATGCACCCTGTAAAAAAAGAAAATGATGTACTGTCTAGGGCGGCGGATT
dxr.TC.KO-r	CTGAAGCCCTACGCTAACAAATAGCGCGACTCTCTGTAGCCGGATTATCCTCTAGGGCGGCGGATT
folA.TC.KO-r	AAGACGCGACCGGCGTCGCATCCGGCGCTAGCCGTAAATTCTATACAAAATCTAGGGCGGCGGATT
nadE.TC.KO-r	CCGGCGTGAACAAATTACTCTTTTTCGCACAATCCAATATGTGCAAATTATCTAGGGCGGCGGATT
pyrH.TC.KO-r	CCCCCGCGATACTTACGCGGAATCTTACCCTTATTTATCCATCACGGGAATCTAGGGCGGCGGATT
adk.TC.KO-r	CTAACCCTCTCCCCGAGGGGGCGAGGGGACTGTCCGTGCGCGCTTTCGAATCTAGGGCGGCGGATT
lpxC.TC.KO-r	AGAGAGTGCCAGATTTGCCAGTCGAATTTTATACGACAGTATAAATGTCGTCTAGGGCGGCGGATT
ribA.TC.KO-r	GCCGGTTATTTTGCTTCCGGCAAGCAAATGAATTACACAATGCAAGAGGGTCTAGGGCGGCGGATT
glnS.TC.KO-r	TTTTAAGTTTCGCTATGCCGGATGGGGCGTTTACGTCGCATCCGGCAAGGTCTAGGGCGGCGGATT
glmS.TC.KO-r	AAAAACATAACAGGAAGAAAAATGCCCCGCTTACGCAGGGCATCCATTTATCTAGGGCGGCGGATT
gmk.TC.KO-r	ATACTTAAAAAGCTCCACAGGTGAAGAAATGACTGGGCATGATACTGAAATCTAGGGCGGCGGATT

Cloning toxins into vector

PemK.f	TAT AGG TAC CAT GAT TGT AAA ACG CGG CGA C
PemK.r	TAT AAA GCT TCT AGA AAT CGA TTA GTC CTA AAC TGA TTT G
Hok.f	TAT AGG TAC CAT GAA ACT ACC ACG AAG TTC CC
Hok.r	TAT AAA GCT TCT ACT TAC CGG ATT CGT AAG CC
PndA.f	TAT AGG TAC CAT GCC ACA ACG AAC GTT TTT AAT G
PndA.r	TAT AAA GCT TTT AAC GTT TAA CTT CGT AGG CTA ACG
PasB.f	TAT AGG TAC CAT GGC TTG GCG GAT TGA ATT TG
PasB.r	TAT AAA GCT TCT AGC GGT ACA CCT CTC GC
VapC.f	TAT AGG TAC CAT GCT GAA ATT CAT GCT TGA TAC C
VapC.r	TAT AAA GCT TTT AGC ACC AGT CTT CGA TTC G
Strp.f	TAT AGG TAC CAT GGG CAT CAC CGG CA
Strp.r	TAT AAA GCT TCT ACA CCT TGG TGA AGG TGT C
FlmA.k1.f	TAT AGG TAC CAT GAA ACT ACC ACG CAG CTC T
FlmA.h3.r	TAT AAA GCT TCT ACT TAC CGG ATT CGT AAG CC
HigB.k1.f	TAT AGG TAC CAT GCA CCT GAT AAC TCA AAA AGC AT

HigB.h3.r	TAT AAA GCT TTC ATT TTT TCC CCT TAG TAC GAT GAA C
MazF.k1.f	TAT AGG TAC CAT GGT AAG CCG ATA CGT ACC C
MazF.h3.r	TAT AAA GCT TCT ACC CAA TCA GTA CGT TAA TTT TGG
YafQ.k1.f	TAT AGG TAC CAT GAT TCA AAG GGA TAT TGA ATA CTC GG
YafQ.h3.r	TAT AAA GCT TTT ACC CAA AGA GCG CCG
YhaV.k1.f	TAT AGG TAC CAT GGA TTT TCC ACA AAG GGT TAA TGG
YhaV.h3.r	TAT AAA GCT TTC AAT GGG TTT CTT CTG TTT CTC G
RnlA.k1.f	TAT AGG TAC CAT GAC AAT CAG GAG TTA CAA AAA CTT AAA TC
RnlA.h3.r	TAT AAA GCT TTC AAA CAA TAT ATA AGT CCT TGA TTA TTC CCC
RelEQ.k1.f	TAT AGG TAC CAT GGC GTA TTT TCT GGA TTT TGA CG
RelEQ.h3.r	TAT AAA GCT TTC AGA GAA TGC GTT TGA CCG
ccdB.kpnI-f	ATT AGG TAC CAT GCA GTT TAA GGT TTA CAC CTA TAA AA
ccdB.hnd3-r	TAT AAA GCT TTT ATA TTC CCC AGA ACA TCA GGT TAA T
K1.endo.1-fB	tataGGTACCatgAGCAACAAGAAGCAGAGC
H3.endo.2-r	tataAAGCTTttaCTTGCTTGTCAGCTGCTC
Constructing additional riboregulator switches	
xho-pARA-f	TATACTCGAGAAGAAACCAATTGTCCAT
PacI-pARA-r	TATATTAATTAAGGTCAGTGCGTCCTGCTGTATGGAGAAACAGTAGAG
xho-pRHA-f	TATACTCGAGTGGCCTCCTGATGTCGTC
PacI-pRHA-r	TATATTAATTAAGGTCAGTGCGTCCTGCTGTACGACCAGTCTAAAAAG
Constructing supplemental repressor cassette	
rrnB.tetR-f	AAAAATAAATGCTTGACTCTGTAGCGGGAAGGCGTATTATGCACACCCCTCTAGAGAAAGACATGACGTACTAGATGTCCA GATTAGATAAA
tetR-r	CTAGTTTGTCCCCTCTTTCTCTAGATCTAGATTAAGACCCACTTTCACA
lacI-f	TCTAGAGAAAGAGGGGACAAACTAGatgAAACCAGTAACGTTA
lacI-r	tcaCTGCCCGCTTTCCAG
ribA.repressors-f	CGTTATGGCAAAATAAGCCAATACAGAACCAGCATTATCTGGAGAATTTCGACGTCATCGATtcaC
ribA.repressors-r	GGTTATTTTGCTTCCGGCAAGCAAATGAATTACACAATGCAAGAGGGAAAAATAAATGCTTGACT
Oligo mutagenesis of *lacI*	
lacI-del	GCGGGCCCATTAAGTTCTGTCTCGGCGCGTCTGCGTCTGGCTGGcATAAATATCTCACTCGCAATCAAATTCAGCC GATAGCGGAACGGG
lacI-ins	GCGGGCCCATTAAGTTCTGTCTCGGCGCGTCTGCGTCTGGCTGGCTGGCtggcATAAATATCTCACTCGCAATCAA ATTCAGCCGATAGCGGAACGGG
lacIq allele	G*A*C TCT CTT CCG GGC GCT ATC ATG CCA TAC CGC GAA AGG TTT TGC ACC ATT CGA TGG TGT CAA CGT AAA TGC ATG CCG CTT CGC CTT CCG
lacIq1 allele	G*A*A TTG ACT CTC TTC CGG GCG CTA TCA TGC CAT ACC GCG AAA GGT GGT GTC AAC GTA AAT GCA TGC CGC TTC GCC TTC CGG CCA CCA GAA

Supplementary Table 2. Table of synthesized gene sequences.

EcoRI Nuclease Pt 1:
ATGAGCAACAAGAAGCAGAGCAACCGCCTGACCGAGCAGCATAAGCTGAGCCAGGGCGTGATTGGCATCTTCGGCGATTACG
CCAAAGCACACGACCTGGCAGTGGGTGAGGTGAGTAAGCTGGTGAAGAAGGCCCTGAGTAACGAGTACCCGCAGCTGAGCTT
CCGTTATCGCGACAGCATCAAAAAAACCGAGATCAACGAGGCCCTGAAGAAGATCGATCCGGACCTGGGCGGCACCCTGTTC
GTGAGCAACAGTAGCATCAAGCCGGACGGCGGCATCGTTGAAGTGAAGGACGACTACGGTGAGTGGCGTGTGGTGTTAGTGG
CCGAGGCCAAGCATCAGGGCAAGGATATCATCAACATCCGCAACGGCCTGCTGGTTGGCAAACGTGGTGACCAAGATCTGATG
GCAGCCGGCAACGCCATCGAGCGCAGCCACAAGAATATTAGCGAGATCGCAAATTTCATGCTGAGCGAGAGCCACTTCCCGTA
TGTGCT

EcoRI Nuclease Pt 2:
CCAAGCATCAGGGCAAGGATATCATCAACATCCGCAACGGCCTGCTGGTTGGCAAACGTGGTGACCAAGATCTGATGGCAGCC
GGCAACGCCATCGAGCGCAGCCACAAGAATATTAGCGAGATCGCAAATTTCATGCTGAGCGAGAGCCACTTCCCGTATGTGCT
GTTCTTAGAGGGTAGTAACTTCCTGACCGAGAACATTAGCATCACCCGTCCTGATGGCCGCGTGGTGAACCTGGAATATAACAG
CGGCATCCTGAATCGCCTGGACCGCCTGACAGCCGCCAACTACGGCATGCCGATCAACAGTAATCTGTGTATTAACAAGTTCG
TTAATCACAAAGACAAGAGCATCATGCTGCAGGCCGCCAGCATCTACACCCAAGGCGACGGCCGCGAGTGGGATAGTAAAATC
ATGTTCGAGATCATGTTTGACATTAGCACAACCAGCCTGCGCGTGTTAGGCCGTGATCTGTTCGAGCAGCTGACAAGCAAGTAA

B anthracis Ames pemK:
ATGATTGTAAAACGCGGCGACGTGTATTTTGCAGACCTTTCCCCAGTTGTTGGTTCTGAGCAAGGAGGTGTTCGTCCGGTTCTT
GTCATTCAAAATGACATCGGAAATCGTTTTAGTCCAACGGTGATTGTAGCGGCTATTACTGCACAGATTCAAAAAGCGAAATTAC
CCACTCATGTGGAAATTGATGCGAAAAAGTACGGTTTTGAGAGAGATTCTGTTATTTTACTTGAGCAGATTCGAACAATCGATAA
GCAGCGCTTAACGGACAAAATCACTCACTTAGATGAAGTGATGATGATTCGTGTAGATGAAGCGCTACAAATCAGTTTAGGACT
AATCGATTTCTAG

E coli O111:H8 pndA
ATGCCACAACGAACGTTTTTAATGATGTTAATCGTCGTCTGTGTGACGATACTGTGTTTTGTCTGGATGGTGAGGGATTCGCTTT
GCGGATTCCGTGTCGAGCAGGGAAACACAGTGCTTGTGGCAACGTTAGCCTACGAAGTTAAACGTTAA

E coli pC15 hok
ATGAAACTACCACGAAGTTCCCTTGTCTGGTGTGTGTTGATCGTGTGTCTCACACTGTTGATATTCACTTATCTGACACGAAAAT
CGCTGTGCGAGATTCGTTACAGAGACGGACACAGGGAGGTGGCGGCTTTCATGGCTTACGAATCCGGTAAGTAG

P fluorescens pasB
ATGGCTTGGCGGATTGAATTTGACCGCGCTGCAGAGCGCGAGCTGGGCAAACTCGACCCGCAAATCGCTAAACGAATCCTGTT
GTTTCTGCATGAGCGGGTATCAAATCTGGATGATCCGCGCAGCATTGGCGAAGCATTAAAAGGCTCACGTTTAGGGGATTTTTG
GAAGTATCGGGTAGGCGACTACCGCCTTATCAGCAGCATCGAGGACGGCGCGTTGCGCATCCTGGTGATTAAGATTGGGAAC
CGGCGAGAGGTGTACCGCTAG

S enterica vapC
ATGCTGAAATTCATGCTTGATACCAATACCTGTATTTTCACCATCAAAAATAAGCCCGAACACATCAGAGAACGCTTCAACCTCA
ATACATCCCGAATGTGTATCAGCTCCATCACCTTAATGGAGCTGATTTACGGTGCTGAAAAAAGCCTGGCGCCGGAGCGTAATC
TTGCCGTCGTGGAGGGATTTATCTCCCGCCTTGAGGTTTTGGATTACGATACACAGGCAGCGATACATACCGGTCAAATCCGTG
CCGAACTGGCCCGCAAGGGAACACCTGTCGGGCCTTATGACCAGATGATTGCTGGCCATGCCGGTAGCCGCGGACTGGTCGT
CGTCACAAACAATCTCCGCGAATTTGAACGCATTCCGGGTATCCGAATCGAAGACTGGTGCTAA

Streptavidin
ATGGGCATCACCGGCACCTGGTACAACCAGCTCGGCTCGACCTTCATCGTGACCGCGGGCGCCGACGGCGCCCTGACCGGA
ACCTACGAGTCGGCCGTCGGCAACGCCGAGAGCCGCTACGTCCTGACCGGTCGTTACGACAGCGCCCCGGCCACCGACGGC
AGCGGCACCGCCCTCGGTTGGACGGTGGCCTGGAAGAATAACTACCGCAACGCCCACTCCGCGACCACGTGGAGCGGCCAG
TACGTCGGCGGCGCCGAGGCGAGGATCAACACCCAGTGGCTGCTGACCTCCGGCACCACCGAGGCCAACGCCGACAAGTCC
ACGGACGACGGCGACGACACCTTCACCAAGGTGTAG

Supplementary Table 3. Calculations for relative fitness of contained and ancestral strains in competitive co-culture. Equations underlying these calculations shown in Materials and Methods.

Dilution Step	Relative Abundance of EcR1rib	Fraction Retained During Step	Avg Doubling Time EcR1rib (mins)	Avg Doubling Time Competitor (mins)
1	0.430	0.791	114	105
2	0.340	0.853	112	107
3	0.290	0.828	113	107
4	0.240	0.975	109	108
5	0.234	0.530	126	105
6	0.124	N/A	N/A	N/A
		**Average:**	**115**	**106**

Supplementary Table 4. Strains used in this paper with genotype, fitness, and escape frequency information.

Strain Name	Growth Requirements	Escape Frequency	Doubling Time (min)	Genotype
**Ancestor Strains**				
MG1655	None	N/A	56 ± 1	
EcNR1	None	N/A	56 ± 1	Δ{ybhB-bioAB}::{λcI857 N(*cro-ea59*)::tetR-bla}
**Single layer (episomal ribo-essential)**				
Ec[R1gmk]	None	1	57 ± 1	EcNR1 gmk::tolC [pLtetO.taRNA pLlacO.cr.gmk]
**Single layer (genomic ribo-essential)**				
EcR1rib	IPTG, aTc	$4.5 \pm 1.0 \times 10^{-6}$	57 ± 1	EcNR1 13B.{pLtetO.taRNA pLlacO.cr.ribA} ribA::tolC
EcR1adk	IPTG, aTc	$3.4 \pm 2.0 \times 10^{-6}$	57 ± 1	EcNR1 13B.{pLtetO.taRNA pLlacO.cr.adk} adk::kanR
EcR1pyr	IPTG, aTc	$1.1 \pm .04 \times 10^{-6}$	67 ± 2	EcNR1 21B.{pLtetO.taRNA pLlacO.cr.pyrH} pyrH::tolC
EcR1glm	IPTG, aTc	$2.0 \pm 0.9 \times 10^{-6}$	56 ± 1	EcNR1 21B.{pLtetO.taRNA pLlacO.cr.glmS} glmS::kanR
EcR1gmk	IPTG, aTc	$5.2 \pm 3.3 \times 10^{-6}$	74 ± 1	EcNR1 21B.{pLtetO.taRNA pLlacO.cr.gmk} gmk::tolC
EcR1acp	None	N/A		EcNR1 21B.{pLtetO.taRNA pLlacO.cr.acpP} acpP::tolC
EcR1nad	None	N/A		EcNR1 21B.{pLtetO.taRNA pLlacO.cr.nadE} nadE::kanR
EcR2nad	Ara, aTc	$1.9 \pm 1.5 \times 10^{-6}$	56 ± 1	EcNR1 13B.{pLtetO.taRNA pLlacO.cr.ribA} 21B.pARA$_{BAD}$.cr.nadE nadE::kanR
EcR3glm	Rha, aTc [1]	$3.8 \pm 0.9 \times 10^{-6}$	57 ± 1	EcNR1 13B.{pLtetO.taRNA pRHA$_{BAD}$.cr.glmS} glmS::kanR
**Two layer (two genomic ribo-essentials, same promoter set)**				
EcR1ribR1rib	IPTG, aTc	$1.4 \pm 0.3 \times 10^{-5}$	56 ± 1	EcNR1 13B.{pLtetO.taRNA pLlacO.cr.ribA} 21B.pLlacO.cr.ribA ribA::tolC
EcR1ribR1adk	IPTG, aTc	$2.6 \pm 2.1 \times 10^{-6}$	59 ± 1	EcNR1 13B.{pLtetO.taRNA pLlacO.cr.ribA} 21B.pLlacO.cr.adk ribA::tolC adk::kanR
EcR1ribR1pyr	IPTG, aTc	$7.4 \pm 5.2 \times 10^{-6}$	68 ± 2	EcNR1 13B.{pLtetO.taRNA pLlacO.cr.ribA} 21B.pLlacO.cr.pyrH ribA::tolC pyrH::kanR
EcR1ribR1glm	IPTG, aTc	$2.3 \pm 0.9 \times 10^{-5}$	58 ± 1	EcNR1 13B.{pLtetO.taRNA pLlacO.cr.ribA} 21B.pLlacO.cr.glmS ribA::tolC glmS::kanR

EcR1ribR1dxr	IPTG, aTc	7.0 ± 2.1 x 10^{-6}	60 ± 1	EcNR1 13B.{pLtetO.taRNA pLlacO.cr.ribA} 21B.pLlacO.cr.dxr ribA::tolC dxr::kanR
EcR1ribR1nad	IPTG, aTc	1.3 ± 1.1 x 10^{-5}	55 ± 1	EcNR1 13B.{pLtetO.taRNA pLlacO.cr.ribA} 21B.pLlacO.cr.nadE ribA::tolC nadE::kanR
EcR1ribR1gmk	IPTG, aTc	9.3 ± 7.8 x 10^{-6}	80 ± 1	EcNR1 13B.{pLtetO.taRNA pLlacO.cr.ribA} 21B.pLlacO.cr.gmk ribA::tolC gmk::kanR
EcR1ribR1lpx	IPTG, aTc	4.2 ± 2.0 x 10^{-6}	56 ± 1	EcNR1 13B.{pLtetO.taRNA pLlacO.cr.ribA} 21B.pLlacO.cr.lpxC ribA::tolC lpxC::kanR
**Single layer enhancements (genomic ribo-essentials)**				
EcR1ribASV	IPTG, aTc	1.2 ± 0.5 x 10^{-5}	60 ± 2	EcNR1 13B.{pLtetO.taRNA pLlacO.cr.ribA.ASV} ribA::tolC
EcR1ribAAV	IPTG, aTc	3.5 ± 2.9 x 10^{-7}	62 ± 4	EcNR1 13B.{pLtetO.taRNA pLlacO.cr.ribA.AAV} ribA::tolC
EcR1rib*lacIq1*	IPTG, aTc	1.4 ± 1.0 x 10^{-6}	55 ± 3	EcNR1 13B.{pLtetO.taRNA pLlacO.cr.ribA} ribA::tolC *lacIq1*
EcR1rib[tetR]	IPTG, aTc	9.9 ± 4.2 x 10^{-8}	60 ± 1	EcNR1 13B.{pLtetO.taRNA pLlacO.cr.ribA} ribA::tolC [pARA$_{BAD}$.tetR]
EcR1rib[lacI]	IPTG, aTc	9.1 ± 7.2 x 10^{-9}	60 ± 1	EcNR1 13B.{pLtetO.taRNA pLlacO.cr.ribA} ribA::tolC [pARA$_{BAD}$.lacI]
EcR1rib+	IPTG, aTc	4.6 ± 3.4 x 10^{-8}	59 ± 1	EcNR1 13B.{pLtetO.taRNA pLlacO.cr.ribA} ribA::p$_{rrnB}$.tetRlacI *lacIq1*
**Bacteriotoxic safeguard layer**				
Ec[Teco]	None	9.4 ± 7.8 x 10^{-7}	56 ± 1	EcNR1 [pARA$_{BAD}$.ecoRI]
Ec[Tpas]	None	4.0 ± 3.6 x 10^{-7}	61 ± 1	EcNR1 [pARA$_{BAD}$.pasB]
EcTeco	None	6.2 ± 1.0 x 10^{-6}	61 ± 2	EcNR1 13B.{pARA$_{BAD}$.ecoRI} 21B.{pARA$_{BAD}$.ecoRI}
EcEAM	aTc	2.4 ± 1.2 x 10^{-6}	60 ± 2	EcNR1 13B.{pLtetO.ecoRImet} 21B.{p434.ecoRInuc}
**Multilayered strains**				
EcR1ribR2nad+	Ara, IPTG, aTc	<3.6 x 10^{-10}	58 ± 2	EcNR1 13B.{pLtetO.taRNA pLlacO.cr.ribA} 21B.pARA$_{BAD}$.cr.nadE nadE::tolC ribA::p$_{rrnB}$.tetRlacI *lacIq1*
EcR1rib[Teco]+	IPTG, aTc	<5.6 x 10^{-10}	61 ± 1	EcNR1 13B.{pLtetO.taRNA pLlacO.cr.ribA} ribA::p$_{rrnB}$.tetRlacI *lacIq1 [pARA$_{BAD}$.ecoRI]*
EcR1ribR3glm[Teco]+	IPTG, aTc, Rha [1]	<3.6 x 10^{-10}	62 ± 1	EcNR1 13B.{pLtetO.taRNA pLlacO.cr.ribA} 910B.{pRHA$_{BAD}$.cr.glmS} ribA::p$_{rrnB}$.tetRlacI glmS::tolC *lacIq1 [pARA$_{BAD}$.ecoRI]*
EcR1ribR3glmEAM+	IPTG, aTc, Rha [1]	<1.3 x 10^{-12}	66 ± 1	EcNR1 13B.{pLtetO.taRNA pLlacO.cr.ribA} 910B.{pRHA$_{BAD}$.cr.glmS} ribA::p$_{rrnB}$.tetRlacI glmS::{pLtetO.ecoR1met} 21B.{specR.p434.ecoR1nuc} *lacIq1*

Legend:

R1 = pLtetO.taRNA pLlacO.cr.Essential gene ORF

R2 = pLtetO.taRNA $pBAD_{ARA}$.cr.Essential gene ORF

R3 = pLtetO.taRNA $pBAD_{RHA}$.cr.Essential gene ORF

+ = p_{rrnB}.tetRlacI lacIq1

taRNA = transactivating RNA

cr = cis-repressed ribosome binding site

{ } = Chromosomal Cassette

ara = arabinose

rha = rhamnose

21B = Safe genomic insertion region, coordinate: 2,428,900

13B = Safe genomic insertion region, coordinate: 1,415,470

[ ] = Plasmid Cassette - All plasmid inserts are between kpnI and hindIII on pZE21

[1] Addition of 1mM Glucosamine improves strain fitness, but is not necessary for viability.

Supplementary Table 5. Essential genes selected as targets for construction of ribo-essential strains. Genes were selected based on criteria including: absence of internal KpnI and Hind3 restriction sites; if part of an operon must be at end so as to avoid polar effects during essential gene knockout; difficulty to complement lost function because of cell-intrinsic function of gene product (*i.e.*, *glnS*) or poor permeability of small molecule product (*i.e.*, *ribA*).

Essential Gene	Function	Class
acpP	Acyl carrier protein	Lipid metabolism
dxr	Isoprenoid synthesis, MEP pathway	
lpxC	Lipid A biosynthesis	
hemA	Porphyrin biosynthesis	Essential cofactor biosynthesis
nadE	Synthesis & salvage of NAD^+	
ribA	First step in riboflavin biosynthesis	
folA	Dihydrofolate reductase – THF synthesis	
pyrH	Uridylate kinase	Nucleotide metabolism
adk	Adenylate kinase	
tmk	Thymidlyate kinase	
gmk	Guanylate kinase	
glnS	Glutaminyl-tRNA synthetase	Translation
glmS	Glucosamine biosynthesis	Aminosugar

Supplementary Table 6. Strain-specific SNPs present in escaping clones.

Strain	Gene	Location (MG1655 Coordinates)	WT	Mutation	Consequence	Feature	Doubling Time ±SD (min)	NCBI Biosample Accession
Escapers from EcR1rib and EcR1gmk								
EcR1rib Revertant 1	lacI	366130	TGCCA	T	Frameshift	Indel	56 ± 1	SAMN0326 6098
	Ins-5	2287247	C	T	R286K	SNP		
EcR1rib Revertant 2	lacI	366130	T	Tgcca	Frameshift	Indel	58 ± 1	SAMN0326 6099
EcR1gmk Revertant 1	lacI	366130	T	Tgcca	Frameshift	SNP	67 ± 1	SAMN0326 6101
	yjfF	4451779	A	C	Synonymous	SNP		
	relA	2910991	T	C	Y227C	SNP		
Escapers from EcR1rib+supplemental lacItetR								
EcR1rib+lacItetR Revertant 1	*No SNPs or small indels called*						71 ± 9	SAMN0326 6103
EcR1rib+lacItetR Revertant 2	crRNA of riboregulator	-29 from ATG	T	Tatttgggtat	Change in crRNA secondary structure	Indel	70 ± 3	SAMN0326 6104
EcR1rib+ lacItetR Revertant 3	*No SNPs or small indels called*						204 ± 31	SAMN03266 05
Escapers from EcR2nad								
EcR2nad Revertant 1	*No SNPs or small indels called*						65 ± 1	SAMN0326 6107
EcR2nad Revertant 2	$pAra_{BAD}$	-76 from transcriptional start	G	A	Promoter change	SNP	64 ± 2	SAMN0326 6108
EcR2nad Revertant 3	araC	70852	C	A	L156I	SNP	63 ± 1	SAMN0326 6109

Escapers from EcR1ribR2nad+								
EcR1ribR2nad+ Revertant 1	araC	70415	T	G	L10R	SNP	75 ± 12	SAMN03266111
	mutS	2857468	T	G	V785G	SNP		
	R1rib	crRBS -30	T	TGGGT	Change in crRNA secondary structure	Indel		
	R1rib	crRBS -34	T	TC	Change in crRNA secondary structure	Indel		
EcR1ribR2nad+ Revertant 2	araC	70841	C	T	A152V	SNP	74 ± 1	SAMN03266112
	stfE	1208825	G		Discontinuity in sequence	Indel		
	R1rib	crRBS -30	T	TGGGT	Change in crRNA secondary structure	Indel		
	R1rib	crRBS -34	T	TC	Change in crRNA secondary structure	Indel		
EcR1ribR2nad+ Revertant 3	araC	70841	C	T	A152V	SNP	74 ± 4	SAMN03266113
	R1rib	crRBS -39	T	TGATCCTACCCACGT	Change in crRNA secondary structure	Indel		
Escapers from EcTeco								
EcTeco Revertant 1	araC	70947	C	A	S187R	SNP	59 ± 2	SAMN03266115
	stfE	1208825	G		Discontinuity in sequence	Indel		
	mutS	2857468	T	G	V785G	SNP		
EcTeco Revertant 2	araC	71162	T	G	L259R	SNP	60 ± 2	SAMN03266116
EcTeco Revertant 3	araC	70999	T	G	C205G	SNP	60 ± 1	SAMN03266117
	araC	70997	T	A	V204D	SNP		
	araC	71006	C	G	S207W	SNP		
	araC	71014	C	A	R210S	SNP		
	stfE	1208825	G		Discontinuity in sequence	Indel		

Escapers from EcEAM								
EcEAM Revertant 1	EcoR1 nuclease	Nucleotide 51 of gene	C	T	Nonsense	SNP	61 ± 1	SAMN03266119
EcEAM Revertant 2	tetR	Nucleotide 158 of gene		IS5	Insertion	Insertion of IS5 transposable element	61 ± 1	SAMN03266120
EcEAM Revertant 3	tetR	Nucleotide 318 of gene		A	Insertion	+1 Frameshift	61 ± 1	SAMN03266121
EcEAM Revertant 4	EcoR1 nuclease	Nucleotide 294 of gene	G	T	D99Y	SNP	61 ± 1	SAMN03266778
EcEAM Revertant 5	tetR	Nucleotide 169 of gene		AA	Insertion	+2 Frameshift	61 ± 1	SAMN03266779

Supplementary Table 7. Toxin genes selected as targets for construction of inducible toxin strains. Genes native to *E. coli* MG1655 were amplified by PCR from the genome. Genes native to other organisms were obtained by codon-optimized chemical synthesis (IDT). These genes were drawn from previously described mRNA interferases, membrane destabilizers, cofactor sequesterers (2), topoisomerase poisons, dsDNA endonucleases (3), or toxin-antitoxin loci (4).

Gene	Class	Function	Host
pemK		Cleaves UAX sites in mRNA	*Bacillus*
vapC		Cleaves tRNA-fMet (initiator)	*Salmonella*
pasB		Cleaves mRNA codons in ribosome A site	*Pseudomonas*
higB		Translation-dependent mRNA cleavage	*Vibrio*
mazF	Ribonuclease	Cleaves upstream of ACA triplets	*E. coli* MG1655
relE		Cleaves mRNA codons in ribosome A site	*E. coli* MG1655
yafQ		Cleaves at Lys mRNA codons	*E. coli* MG1655
rnlA		23S rRNA cleavage, some mRNAs	*E. coli* MG1655
yhaV		Degrades rRNA (16S & 23S)	*E. coli* MG1655
flmA		Pore-forming protein	*E. coli* MG1655
pndA	Membrane disruptor	Pore-forming protein	*E. coli* R plasmid
hok		Pore-forming protein	*E. coli* pC15 plasmid
ccdB	Topoisomerase poison	Inhibits gyrase	*E. coli* O157
ecoRI	Deoxyribonuclease	Cleaves at GAATTC sites	*E. coli*
strp	Cofactor sequestration	Binds streptavidin	*Streptomyces*

SUPPLEMENTARY REFERENCES

1. Elena, S.F. and Lenski, R.E. (2003) Evolution experiments with microorganisms: the dynamics and genetic bases of adaptation. *Nature reviews. Genetics*, **4**, 457-469.
2. Szafranski, P., Mello, C.M., Sano, T., Smith, C.L., Kaplan, D.L. and Cantor, C.R. (1997) A new approach for containment of microorganisms: dual control of streptavidin expression by antisense RNA and the T7 transcription system. *Proceedings of the National Academy of Sciences of the United States of America*, **94**, 1059-1063.
3. Yamaguchi, Y., Park, J.H. and Inouye, M. (2011) Toxin-antitoxin systems in bacteria and archaea. *Annu Rev Genet*, **45**, 61-79.
4. Smith, A.S. and Rawlings, D.E. (1998) Efficiency of the pTF-FC2 pas poison-antidote stability system in Escherichia coli is affected by the host strain, and antidote degradation requires the lon protease. *Journal of bacteriology*, **180**, 5458-5462.
